# The REtirement in ACTion exercise programme and its effects on elements of long term functionality in older adults

**DOI:** 10.3389/fpubh.2023.1151035

**Published:** 2023-07-28

**Authors:** Peter Ladlow, Max J. Western, Colin J. Greaves, Janice L. Thompson, Janet Withall, Jolanthe de Koning, Jessica C. Bollen, Sarah J. Moorlock, Jack M. Guralnik, Kenneth R. Fox, Afroditi Stathi

**Affiliations:** ^1^Department for Health, Faculty of Humanities and Social Sciences, University of Bath, Bath, United Kingdom; ^2^Academic Department of Military Rehabilitation (ADMR), Defence Medical Rehabilitation Centre (DMRC) Stanford Hall, Loughborough, United Kingdom; ^3^School of Sport, Exercise and Rehabilitation Sciences, College of Life and Environmental Sciences, University of Birmingham, Birmingham, United Kingdom; ^4^College of Medicine and Health, University of Exeter, Exeter, United Kingdom; ^5^Birmingham Clinical Trials Unit, University of Birmingham, Birmingham, United Kingdom; ^6^Department of Epidemiology and Public Health, School of Medicine, University of Maryland, Baltimore, MD, United States; ^7^Centre for Exercise, Sport and Health Science, University of Bristol, Bristol, United Kingdom

**Keywords:** ageing, mobility disability, physical activity, community, strength, balance

## Abstract

**Background:**

The prevention of mobility-related disability amongst adults is a global healthcare priority. Cost-effective community-based strategies to improve physical function and independence in older adults with mobility limitations are needed. This study investigated the effectiveness of the REtirement in ACTion (REACT) exercise intervention on individual markers of physical function at 6-and 12-months.

**Methods:**

The REACT multicentre randomised controlled trial assigned 777 older adults (female, 514; male 263) (mean age 77·6 [SD 6·8] years) with reduced lower limb physical functioning (Short Physical Performance Battery [SPPB] score 4–9) to receive brief healthy ageing advice or a 12-month, group-based, multimodal exercise programme delivered in local communities. Estimated differences in the three individual component scores of the SPPB (strength, balance, gait speed) and physical functional outcomes recorded at 6- and 12-months were assessed.

**Results:**

The intervention group demonstrated significant improvements in strength (OR = 1.88, 95% CI = 1.36–2.59, *p* < 0.001) and balance (OR = 1.96, 95% CI = 1.39–2.67, p < 0.001) at 12-months, but not in gait speed (OR = 1.32, 95% CI = 0.91–1.90, *p* = 0.139). In comparison to the control group, at six-and 12-months, the intervention group reported statistically significant improvements in Mobility Assessment Tool-Short Form (MAT-SF), physical component score from SF-36 questionnaire, and strength and endurance items of subjectively reported physical activity (PASE 10-item). Greater than 75% adherence (attending ≥48 of the 64 exercise sessions delivered in 12-months) was associated with superior functional outcomes.

**Conclusion:**

The REACT exercise programme provides local, regional and national service providers with an effective solution to increase muscle strength and balance in older adults at risk of mobility disability.

## 1. Introduction

Physical inactivity is one of the strongest predictors of mobility-related disability in older adults (1). Unfortunately, older adults are the least active segment of the United Kingdom population. Less than 30% of 65–74 year-olds report any moderate-intensity physical activity (PA) lasting at least 10 min in the previous 4 weeks (2). However, clinical trials have provided robust evidence that physical components of frailty, such as reduced muscular strength or cardiorespiratory fitness, can be reversed, or at least the progression of these frailty indicators can be slowed, by undertaking an appropriate exercise programme (3–5).

The preservation of physical function in older adults is a major public health priority. It is therefore imperative that healthcare and exercise professionals acquire clear guidance to promote, refer, prescribe, and deliver evidence-based PA interventions to individuals at risk of mobility-related disability. Most current PA guidelines globally recommend engaging in some form of muscle strengthening activity at least twice per week but they lack specific guidance necessary for exercise prescription for older adults (6–9). The US-based National Strength and Conditioning Association (NSCA) provides more detailed information regarding intensity of muscle strengthening activity, generally recommend lifting loads at 70–85% of 1 repetition-maximum (RM) using free weights or machine-based exercises. They also advise power/explosive training using loads that reflect 40–60% of 1RM and from a functional movement perspective, the prescription of exercises that mimic tasks of everyday living (10). However, a significant proportion of these recommendations rely on expensive infrastructure and resources (exercise equipment) not available for the majority of local councils/charities which are trying to deliver cost-effective programmes for improving the long-term health of older adults in their communities.

Whilst successful, previous long-term exercise interventions for older adults at risk of mobility disability describe a lack of functional progression or training specificity to activities of daily living within their strengthening-based exercise programmes (11, 12), increasing the likelihood of reaching a functional plateau and/or training monotony during the course of their intervention (10). Other large multi-centre trials focused on improving general health of older adults describe the strength and balance related activities vaguely, with little to no rationale presented as to why such exercises were selected or progression of the intensity of load delivered (13, 14). Collectively, this makes the dissemination of research findings into real world practise challenging as exercise protocols cannot be replicated or adapted to meet the infrastructure/resource and functional demands of the population of interest. Any cost-effective exercise intervention (15) found to improve derivatives of strength, aerobic capacity, coordination and balance, with positive prospects for sustained health and independence for older adults at risk of mobility disability (16) should therefore be comprehensively evaluated.

The REtirement in ACTion (REACT) study represents the first large-scale (777 participants) pragmatic, trial in the United Kingdom to target the non-disabled but high-risk segment of the older population with an intervention to reduce mobility-related disability in a real-world community setting (15–18). Age-related reductions in muscular strength and muscle mass tends to be more pronounced in the lower versus upper limbs (19). Lower-limb weakness can compromise the ability to perform many activities of daily living, leading to a loss of functional independence and increased risk of falls resulting in injury (20). A reduced ability to walk can predict future disability and older adults who have difficulty walking subsequently have a greater risk for mortality (21). Clear clinical reasoning relating to appropriate progression and regression of exercise is essential to achieve positive functional outcomes (22). The ideal progression model to use with an older adult is one in which there is a smooth increase in loading intensity that optimises strength gains whilst preserving interest levels and enjoyment (23). In order to facilitate continual neuromuscular adaptations over a 12 month intervention, a variety of exercises, and therefore opportunities to add progression (or regression if required) is warranted.

**Figure 1:**
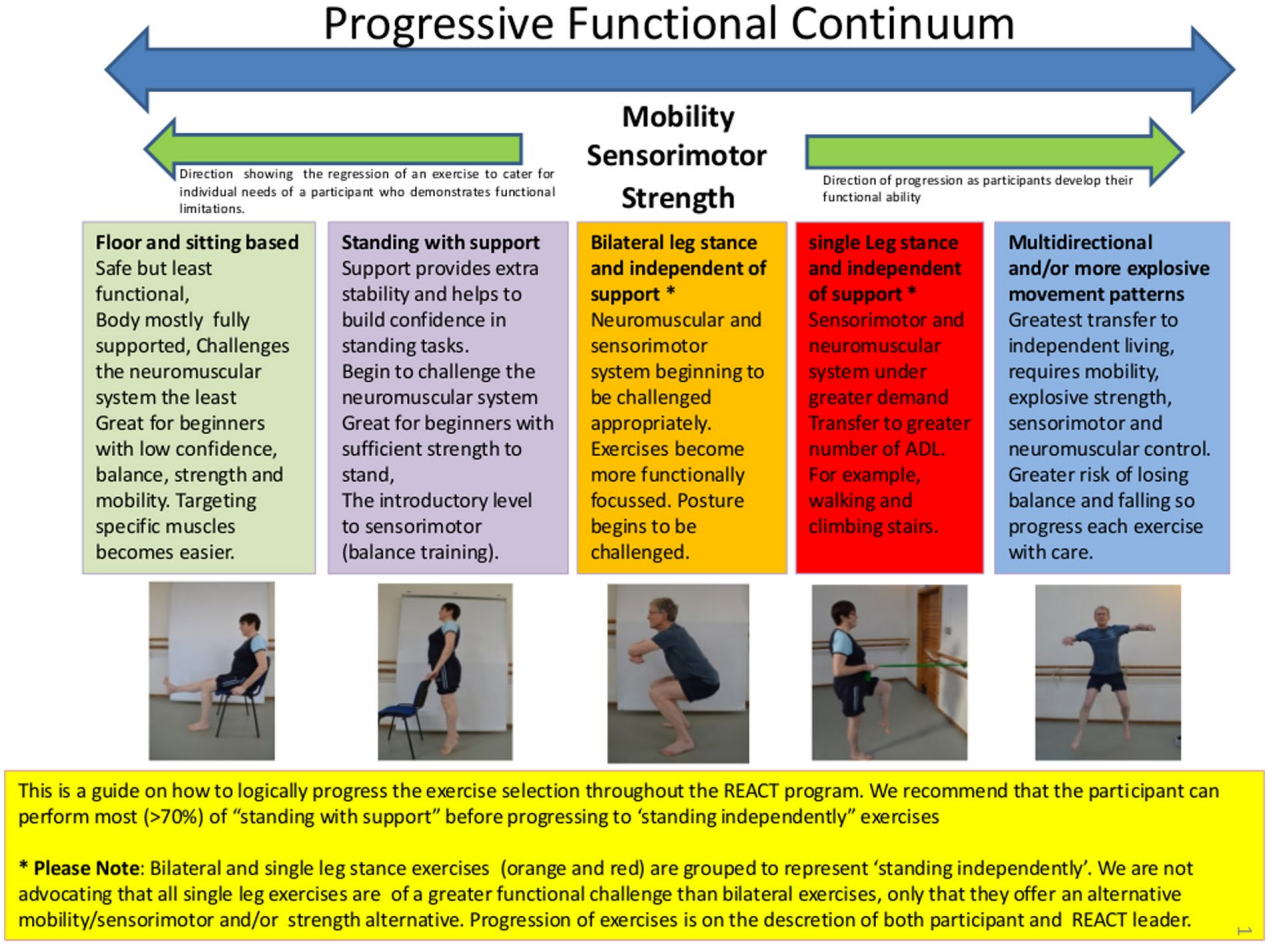
The REACT study progressive functional continuum.

**Figure 2:**
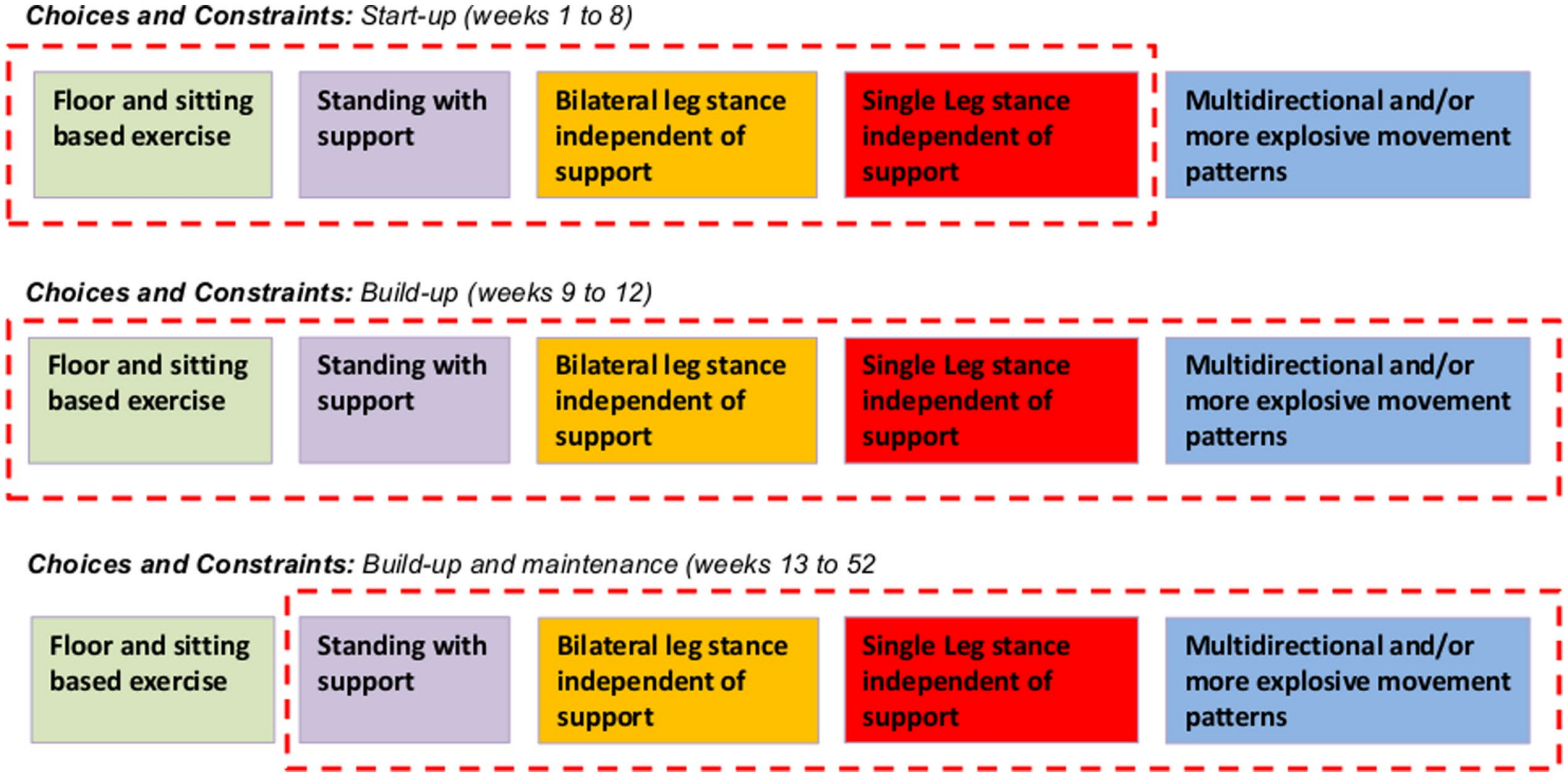
Choices and constraints: transitioning through the functional continuum as time and physical function progress. The choices and constraints model provides exercise leaders with a clear understanding of what type of exercises to prescribe during the supervised sessions during different phases of the overall REACT intervention. To optimise enjoyment and adherence during weeks 1 to 8 (start-up), the prescription of chair-based exercises will be permitted alongside standing with and without support (purple, orange and red blocks). During the first 8 week period, multidirectional and or explosive movements will not be permitted. The model reflects this “constraint” between weeks 1 to 8 by excluding the “multidirectional and or more explosive movement patterns” (blue block) from within the red dotted box. During weeks 9 to 12 (build-up) there are no “constraints” with regards to exercise prescription (depicted in the model by all types of exercise being included within the red dotted box). This does not mean that all participants should be doing explosive movements. Only those individuals who have proven during their supervised sessions to be functionally capable of progressing to these higher function tasks (able to perform many of the unsupported standing exercises independently) may start to perform these activities under the supervision of the exercise leader. From week 13 onwards (incorporating build-up and maintenance phases) constraints will be placed on chair based exercises (depicted in the model by its exclusion from within the red dotted box). This is to reinforce to the exercise leader that all eligible participants should be capable of performing exercises in a standing position (with support) following 3 months of supervised exercise provision. However, it is important to recognise that an element of common sense is required at all times when supervising exercise delivery of older adults at risk of mobility disability. If a participant feels fatigued or has joint pain during a specific task that can be alleviated by selecting a chair-based exercise, then using a chair should be encouraged. Additional information is provided in Supplementary File S1.

**Figure 3:**
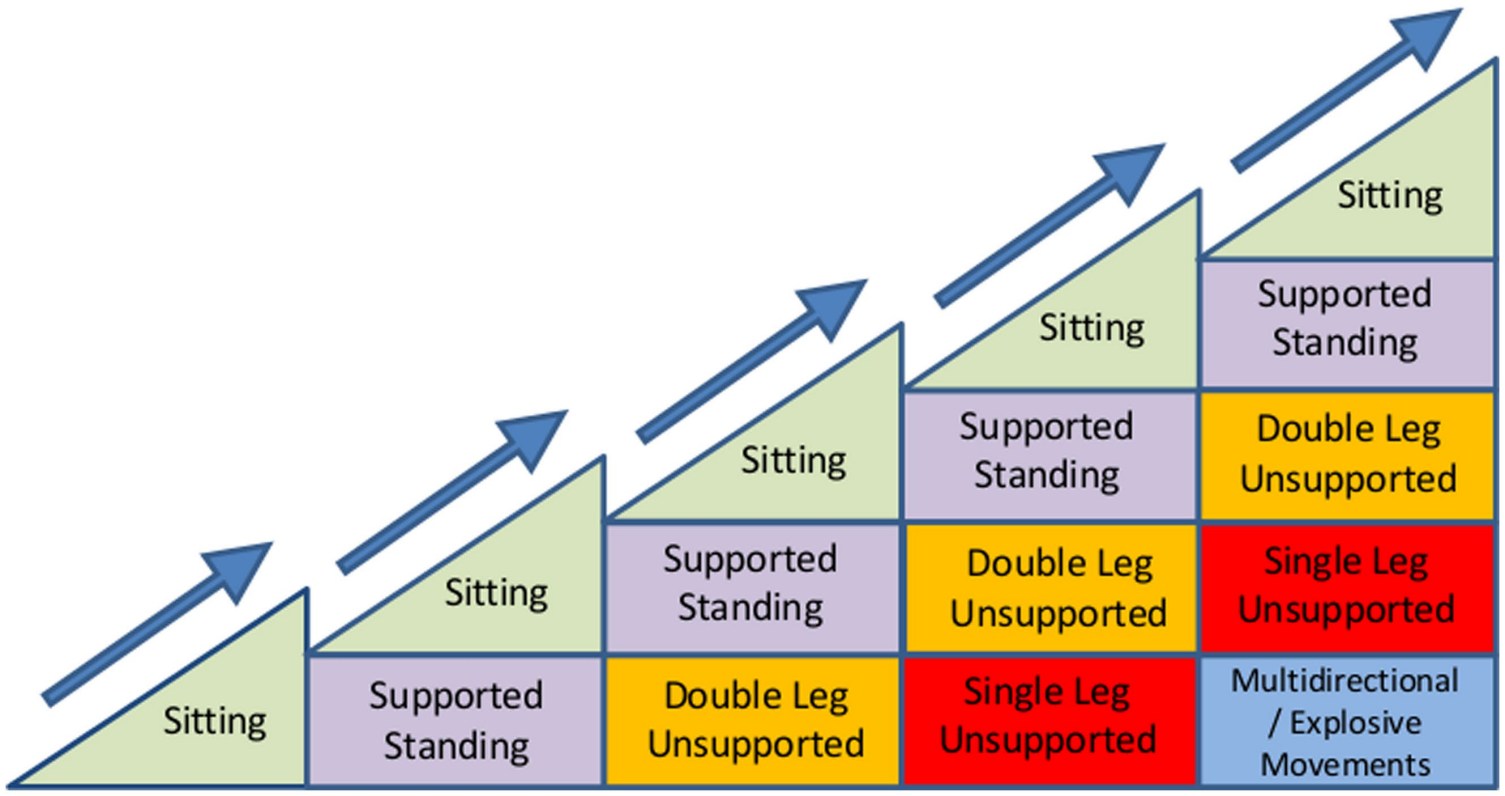
Theoretical model of exercise progression using component of the REACT Functional Continuum. The progression models presented here aim to simplify the reasoning process behind the prescription of exercises and may be applied to any given exercise prescribed within the local community settings. The model aims to help exercise leaders visualise the gradual progression of function or of any single exercise (Figures 4A–C) and to clinically reason the progressions within that exercise. The horizontal axis represents time and the vertical axis the level of functional difficulty. In theory, as the participant adapts to training and becomes more functionally proficient, this increases the number of exercises available to support continual progress and facilitate the individual achieving their functional goals. The number of stages along the horizontal axis and number of progressions along the vertical axis are virtually unlimited and are at the discretion of the REACT exercise leader.

**Figure 4:**
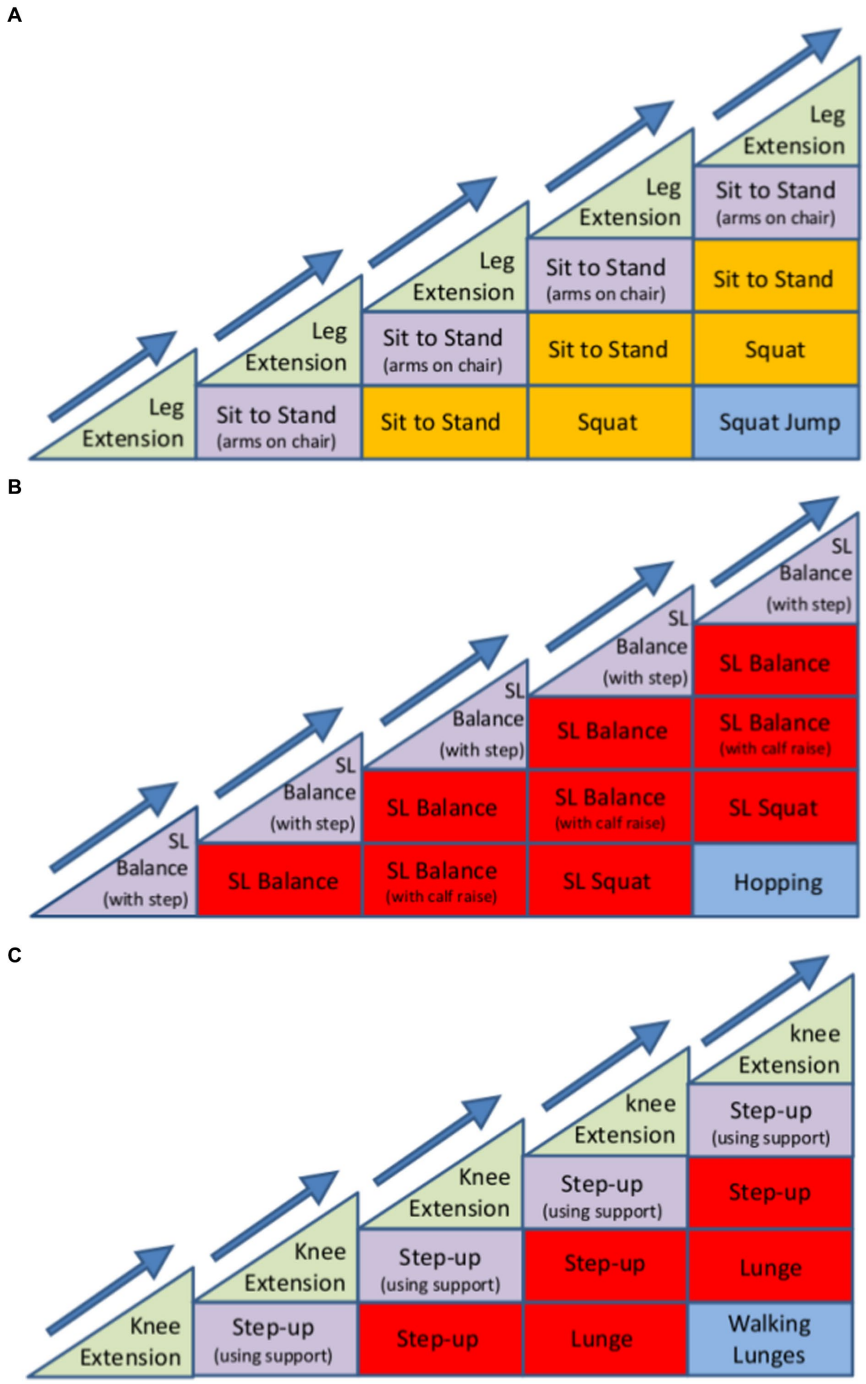
Example of how the REACT progression model can be used to progress the development of **(A)** bilateral lower-limb strength, **(B)** lower limb sensorimotor control (balance), and **(C)** single-limb strength. The model aims to help exercise leaders visualise the gradual progression of any single exercise and to clinically reason the progressions between exercise sessions. Each exercise may be progressed by manipulating a number of variables, including sets, repetitions, speed of contraction, time under tension, base of support, centre of mass, visual aids (eyes open or shut), and the use of external factors (catching an object whilst maintaining balance). The introduction of a new exercise stimulus (new progression), can be interchanged with one another without causing a dramatic progression in the difficulty of the overall exercise. The number and order of exercise progressions is dependent on the individual’s response to exercise and any limiting factors (for example, osteoarthritis causing pain within the joint during specific movements). Therefore, in theory, the number of stages along the horizontal axis and number of progressions along the vertical axis are virtually unlimited and are at the discretion of the REACT exercise leader.

For REACT, a multimodal exercise prescription using primarily callisthenics (exercises that use body weight to generate resistance) of varying functional difficulties was adopted using the “functional exercise continuum” (Figure 1). Each of the 5 categories (exercises grouped by functional demand) within this continuum (sitting, supported standing, double leg unsupported, single leg unsupported, multidirectional/explosive movement) had between 10 and 30 exercises available to help guide the exercise practitioner’s decision making process around exercise prescription/regression. A central theme of the REACT programme was to offer strengthening exercises in standing-based postures which over the short to medium term minimise any reliance on performing exercises whilst sat on a chair. The REACT programme facilitates this by using a “choices and constraints” model (Figure 2). Inspired by Blanchard and Glasgow’s *“Theoretical model for exercise progression as part of a complex rehabilitation programme design”* (24), the REACT progression model (Figure 3) incorporates the unique components of the REACT functional continuum. Figure 4 demonstrates how individual components of the REACT model can by progressed using exercise examples. The number of sets and repetitions prescribed remained largely consistent. The primary principle manipulated to provide training overload was the functional difficulty/intensity of the exercise being prescribed (see Figures 1, 3). Exercise progression over the duration of the intervention was determined using a combination of the participant’s subjective rate of perceived exertion (RPE) and number of repetitions in reserve (Supplementary File S1, Section 3.7.3).

REACT had a low attrition rate (19% at 24 months) and good intervention adherence (18). When compared to an age matched control group, this 12-month group-based exercise and behavioural maintenance intervention, Cross et al. (25) demonstrated improved physical functioning (as determined using the Short Physical Performance Battery [SPPB]), with benefits that were sustained for at least 24 months. After adjustment for baseline SPPB score, age, sex, study site, and exercise group, SPPB scores were significantly higher in the intervention group than in the control group at 6 months (adjusted mean difference 0·68 [95% CI 0·39–0·96]; *p* = 0·0009), 12 months (0·77 [0·40–1·14]; *p* = 0·0010) and 24-month follow-up (0·49 [95% CI 0·06–0·92]; *p* = 0·014). Importantly, a higher intervention effect at 24 months follow-up was associated with increased attendance to the programme of sessions (16).

Whilst the SPPB composite score is useful as a global indicator of physical function, to facilitate future exercise guidelines for older adults it is important to evaluate how each individual component measure of the SPPB (balance, sit to stand and gait speed) responded to this successful 12 month intervention. Furthermore, understanding how each component of the SPPB responded to the REACT programme, alongside other secondary functional outcome measures, will provide greater clarity on the mechanisms underpinning the changes demonstrated in physical function. Such an evaluation will enable recommendations for future exercise programmes delivered in community settings for older adults at risk of mobility disability. Therefore, the aim of this study was to investigate the effectiveness of the REACT programme on the individual markers of lower-limb function as assessed by the three components of the SPPB and secondary outcomes of physical function at 6 months and 12 months follow-up (the supervised element of the REACT intervention) compared to controls.

## 2. Materials and methods

### 2.1. Study design

REACT was a pragmatic, multi-centre, two-arm, parallel-group, single blind, randomised controlled trial (15–18). Ethical approval was provided by the National Health Service (NHS) South East Coast - Surrey Research Ethics Committee (15/LO/2082). This trial is registered with ISRCTN, ISRCTN45627165. The full REACT study protocol has been published elsewhere but without detailed information on the methods used to deliver the exercise component of the trial so that it can be replicated / adapted in the future (17). A summary of the REACT exercise intervention is provided below. A detailed description has been provided as Supplementary File. A CONSORT flow diagram is provided in the REACT main study findings article (16).

### 2.2. Setting

To enable recruitment of a socio-economically and ethnically diverse sample, REACT programme were delivered inside community and leisure centres in both urban and semi-urban communities across England: Bath, Bristol, Birmingham and Devon (17). To ensure the REACT study could be delivered at cost and at a large scale, group exercise sessions were delivered in local community (village hall) and leisure centres (studio space) during off-peak hours. Sessions were organised as group activities with up to 15 participants per group. There was no access to fixed resistance machines, free-weights or cardiovascular machines during supervised sessions. However, each centre location ensured that chairs were provided for participants to use if/when required and elastic therabands and ankle weights were provided by the REACT research team to be used for resistance training.

### 2.3. Participants

Full eligibility criteria for participants are detailed in Table 1. Briefly, community dwelling adults aged 65 years or older who were not in full-time employment and scored between 4 and 9 (inclusive) on the SPPB were recruited. This is based on data showing that older adults with SPPB scores of 9 or less have substantially higher risk of major mobility disability 3 years later, compared with those with a score of 12 (4, 26). The SPPB criteria identified people who have mobility limitations but are still ambulatory, and included people classed as physically frail (SPPB 4–7) and pre-frail (SPPB 8–9) by the European Medicines Agency (27). The eligibility criteria resulted in a participant population with a diverse medical history (see Table 2). Due to the broadness of the inclusion criteria, the exercise protocol had to be inclusive towards a variety of existing chronic health conditions, requiring modifications to traditional exercise prescription to meet the functional needs of the individual. Written informed consent was obtained from all study participants and all experimental procedures correspond to the declaration of Helsinki.

**Table 1:** Eligibility criteria for participation in the REACT study.

Inclusion criteria	Aged 65 years or older and not in full-time employment Planning to reside in the target area (Bath/Bristol, Devon, Birmingham) for at least 24 months Score between 4 and 9 (inclusive) on the Short Physical Performance Battery (SPPB)
Exclusion criteria	Self-reported inability to walk across a room without a walker or the help of another person Existing major mobility limitation (defined as SPPB of 3 or less, or unable to complete the 4-m walk component of SPPB) Living in residential or nursing care Inability to attend the REACT physical activity sessions as scheduled A documented or patient-reported medical condition that would preclude participation, including: • Arthritis so severe it would prevent participation in physical activity • Parkinson’s disease or diagnosed dementia • Any terminal illness • Lung disease requiring use of orally administered corticosteroids or supplemental oxygen • Severe kidney disease requiring dialysis • Severe heart disease that would prevent participation in physical activity (e.g., chest pain when walking 100 or 200 yards or up a flight of stairs) • Implanted cardiac defibrillator • Cardiac arrest which required resuscitation • Severe uncontrolled psychiatric illness • Currently receiving radiation therapy or chemotherapy treatment for cancer • Awaiting knee or hip surgery • Major heart surgery (including valve replacement or bypass surgery) in the last 6 months • Unstable heart condition (e.g., uncontrolled arrhythmia, angina, heart failure or hypertension) • Spinal surgery in the last 6 months • Any other clinical condition that the person’s GP or clinician considers would make them unsuitable for participation in a physical activity rehabilitation programme to prevent decline of lower-limb functioning
Temporary exclusion criteria	Heart attack (or myocardial infarction), stroke, spinal surgery, hip fracture, hip or knee replacement within the previous 6 months Currently receiving physical therapy on legs or enrolled in another physical activity research or intervention study

**Table 2:** Baseline characteristics of trial participants.

	Intervention group (*n* = 410)	Control group (*n* = 367)
Age, years	77·8 (6·93)	77·3 (6·64)
Sex		
Female	273 (67%)	241 (66%)
Male	137 (33%)	126 (34%)
Ethnicity		
Caucasian or White	387 (94%)	352 (96%)
African or Caribbean	14 (3%)	9 (2%)
Asian	5 (1%)	4 (1%)
Other or mixed	4 (1%)	2 (1%)
Highest education level		
Less than secondary	31 (8%)	33/366 (9%)
Completed secondary	142 (35%)	154/366 (42%)
Some college or vocational training	117 (29%)	89/366 (24%)
College or university degree	89 (22%)	73/366 (20%)
Graduate degree or higher	31 (8%)	18/366 (5%)
Index of multiple deprivation		
Quintile 1	43 (10%)	43 (12%)
Quintile 2	83 (20%)	73 (20%)
Quintile 3	89 (22%)	70 (19%)
Quintile 4	82 (20%)	74 (20%)
Quintile 5	113 (28%)	107 (29%)
Existing medical condition^a^		
Coronary heart disease	20 (5%)	18 (5%)
Atrial fibrillation	43 (11%)	32 (9%)
Peripheral arterial disease	4 (1%)	5 (1%)
Stroke/TIA	30 (7%)	20 (6%)
Hypertension	198 (49%)	159 (44%)
Type 2 diabetes mellitus	59 (15%)	53 (16%)
Chronic kidney disease	6 (1%)	6 (2%)
Rheumatoid arthritis	31 (8%)	30 (8%)
Osteoarthritis	90 (22%)	64 (18%)
Osteoporosis	49 (12%)	35 (10%)
Asthma	43 (11%)	32 (9%)
COPD	28 (7%)	21 (6%)
Cancer	24 (6%)	13 (4%)

Data are reported as mean (SD) or *n* (%); SPPB, Short Physical Performance Battery; TIA, Transient Ischaemic Attack; COPD, Chronic Obstructive Pulmonary Disease.

a These values represent the numbers (and percentages) from a total of 404 participants in the intervention groups and 360 participants in the control group.

### 2.4. Recruitment

In brief, the vast majority of participants were recruited from 35 primary care practises across England. This was driven by invitation letters from general practitioners (GPs) as well as advertising by third sector or charity organisations, local media (articles and low-cost advertising in local newspapers, magazines, radio, and at community events), and word of mouth. A detailed account of the recruitment strategy and actions to meet the recruitment targets have been published separately (28).

### 2.5. Randomisation and blinding

The randomisation and blinding process has been described in detail elsewhere (16, 17). In brief, REACT used a 1:1 randomisation strategy stratified by baseline SPPB score, age, sex, and study site. Similar to other studies of behavioural interventions, masking of the participants to study group was not possible, which introduces the possibility of social desirability bias in secondary patient-reported outcome measures. However, the primary outcome consisted of a battery of physical performance tasks assessed by independent observers with the data collectors masked to study group allocation (18). The trial statistician was blinded throughout.

### 2.6. The REACT exercise intervention

The overall structure of the REACT study intervention (including the behavioural maintenance element) is described elsewhere (16–18, 25). However, a brief description of the overall 12 month REACT intervention is described in Table 3.

**Table 3:** The REACT study: generic structure (Week 1 to week 52).

*Start-up (adoption: weeks 1 to 8)* *T*he purpose of this phase is to stimulate initial increases in PA and fitness, to reduce any anxieties or concerns about exercise, and to build confidence and a sense of attachment to the programme Each participant will receive a 45-min individualised, face-to-face introductory session which will be used to personalise the programme for starting levels and progression Two 60-min PA sessions per week, plus 15–20 min of social time, will then be delivered by the REACT trainer
*Build-up (adoption: weeks 9 to 24)* A 45-min interactive educational/social session run by the REACT trainers will be added at the end of one of the two weekly sessions. These sessions will use evidence-based, person centred behaviour-change strategies to build intrinsic motivation and self-efficacy. They will be designed to maximise enjoyment, social interaction and group identity. Behavioural management will focus on self-regulation using goal setting, self-monitoring, reviewing of goals and problem-solving. A key focus will be on exploring and planning transition to more lifestyle-based activities Pedometers will be introduced during these sessions to support the participant in the transition to the maintenance phase After week 12, the exercise session frequency is reduced to once per week but with an expectation that participants find an hour per week to exercise at home, in the neighbourhood or at a PA session in their local community
*Taking charge (maintenance: weeks 25 to 52)* The maintenance stage will focus further on home and neighbourhood-based activities whilst continuing with a weekly centre-based PA session followed by a short social session Supervised exercise frequency of once per week remains Participants will enact action plans for PA outside of the REACT programme that were made during the transition phase and will be supported through group social/education meetings once a month Encourage groups to self-organise their own social interaction beyond the scope of the study and to consider doing activities together Participants will be informed about local opportunities for PA in their community

To support the need for detailed information and clarity about the details of a successful programme we have provided a detailed account of how the REACT exercise intervention was delivered in a comprehensive Supplementary file (see Supplementary File S1). Topics described in Supplementary file: (1) the progressive functional continuum, (2) rational for the inclusion of different methods of exercise (explosive multidirectional movements, upper limb exercises and home-based exercise prescription), (3) general principles adopted for exercise progression, (4) determining the exercise selection for the first exercise session, (5) structure of each exercise session, (6) how the exercise programme was personalised to meet the functional requirements of the individual, (7) how the exercise leader monitored and progressed exercise intensity, (8) when to increase the intensity/functional demands of the exercise and (9) how the exercise programmes meets existing national and international PA and exercise guidelines for older adults.

### 2.7. Control group

Participants allocated to the control group were invited to attend three 60-to-90-min group-based workshops on topics relating to healthy ageing (e.g., healthy eating, dealing with dementia and volunteering) with no physical activity content/intervention. These consisted of presentations and discussion groups on various aspects of healthy ageing and incorporate socialising opportunities.

### 2.8. Outcome measures

Outcome measures relating to physical function were collected at three time points; baseline, 6 months (midway through intervention) and 12 months follow-up (end of intervention). All functional outcomes are fully described in the protocol (17). The primary outcome measure was the SPPB. The SPPB is a summary lower-limb based functional performance measure consisting of 4 metre gait speed at usual pace, a timed repeated chair rise test involving 5 repetitions of sit-to-stand [and back to sit] as quick as possible from a seated position, and three increasingly difficult 10-s standing balance tasks, namely feet together, semi-tandem and full tandem (29). Each performance measure is assigned a categorical score ranging from 0 (inability to complete the task) to 4 (best performing). A summary score ranging from 0 (worst performers, unable to complete) to 12 (best performers) is calculated by summing the three component scores. For the gait speed and chair rise test, the time taken to walk 4-metres at normal walking pace and the time taken to complete the 5-repetitions of sit to stand were recorded and used in the present study as continuous outcomes. Therefore, to better understand the direct and immediate influence of the exercise programme on physical function, this article will only report outcome measures collected during the 12 month supervised component of the REACT intervention.

Secondary subjective functional outcomes measures included the Mobility Assessment Tool-Short Form (MAT-SF) (30), which is a video animated tool that asks participants to rate their ability to complete ten ambulatory-based mobility tasks of varying functional difficulty on a discrete scale. The possible range of scores is from 30 to 80. The MAT-SF has been found to have excellent test–retest reliability (intraclass correlation coefficient = 0.93) and validity (31, 32). The physical component score (PCS) of the SF-36 questionnaire (33) is scored using likert scales and yes/no options based on four scales; physical functioning (10 items), role-physical (4 items), bodily pain (2 items), and general health (5 items). Scores range from 0 to 50 for the PCS with higher scores indicate better health status (33). The SF-36 has reported reliability of 0.81–0.88 (34). The strength and endurance item of the Subjective PA (PASE 10-item questionnaire) (35). PASE uses frequency, duration, and intensity level of activity over the previous week on a scale of “never,” “seldom” (1–2 days), “sometimes” (3–4 days) or “often” (5–7 days). PASE assigns a score, with higher scores indicating greater PA (36). Finally, the Falls Inventory (37) which asks participants to indicate the number of falls they have had in the past 6 months.

### 2.9. Statistical analysis

The analytical models used in the present study mirrored that of the pre-specified analysis of the principal REACT study (16). Sample size was determined for the main REACT study based on the primary outcome of SPPB. Each ordinal (SPPB sub-components, muscle strength exercise frequency), binary (falls), and continuous (4-metre walk time, chair rise time, MAT-SF score, SF36-physical component score) outcome was analysed separately at 6 and 12 months. Models were adjusted for their baseline values of each outcome, the four stratification variables (baseline total SPPB score, age, sex, and study site) and for clustering by exercise group within the intervention group, whilst control group participants were entered as individual groups, each of size one. The logistic and linear mixed models were implemented in Stata SE (version 17.0) using the ‘logit’ and ‘mixed’ commands. Margins from the models were calculated as a probability of scoring in the top two highest categories for ordinal outcomes, and the adjusted mean score for continuous outcomes. Interactions between the group allocation and age (65–74 vs. 75+ years), sex (male vs. female), and baseline physical function (SPPB 4–7 vs. 8–9) were explored to determine if any further stratification of the analysis would yield insights into variations in benefit in relation to these population characteristics. A series of univariate General Linear Models controlling for baseline score of each outcome was used to test the impact on adherence on the mean scores between high (>75%) and low (<75%) adherers for each SPPB ordinal variable. Descriptive analyses were also used to examine the association between session attendance and individual SPPB sub component scores at both six and 12 month assessments, in the intervention group only.

A full report of all severe adverse events (SAEs) is recorded in the main REACT study article (Supplementary Table S7) (16) and in the full National Institute of Health Research (NIHR) report (Table 24) (18). Here, the safety of the exercise intervention were assessed based on the number of SAEs considered to be ‘directly’, ‘probably’ or ‘possibly’ related to the supervised delivery and management of the REACT programme.

## 3. Results

Table 2 displays the baseline participant characteristic of the REACT study population, split by intervention (n, 410) and control group (n, 367). Of the 777 participants were randomised at baseline, 659 (85%) completing the primary SPPB outcome at 6 months and 649 (84%) at 12 months. Of the 410 participants allocated to the intervention group, 66 (16%) did not attend any intervention sessions (non-starters), 78 (19%) attended less than 50% of the sessions offered, 82 (20%) attended 50–74% of sessions, and 138 (45%) attended 75% or more. For participants in the intervention group who engaged with the programme (starters only), the mean percentage of exercise sessions attended was 67.7% (65.1–70.4), or 43.3 h (41.7–45.1 h) of an available maximum of 64 h of supervised exercise sessions offered.

### 3.1. Differences between intervention and control

**Figure 5:**
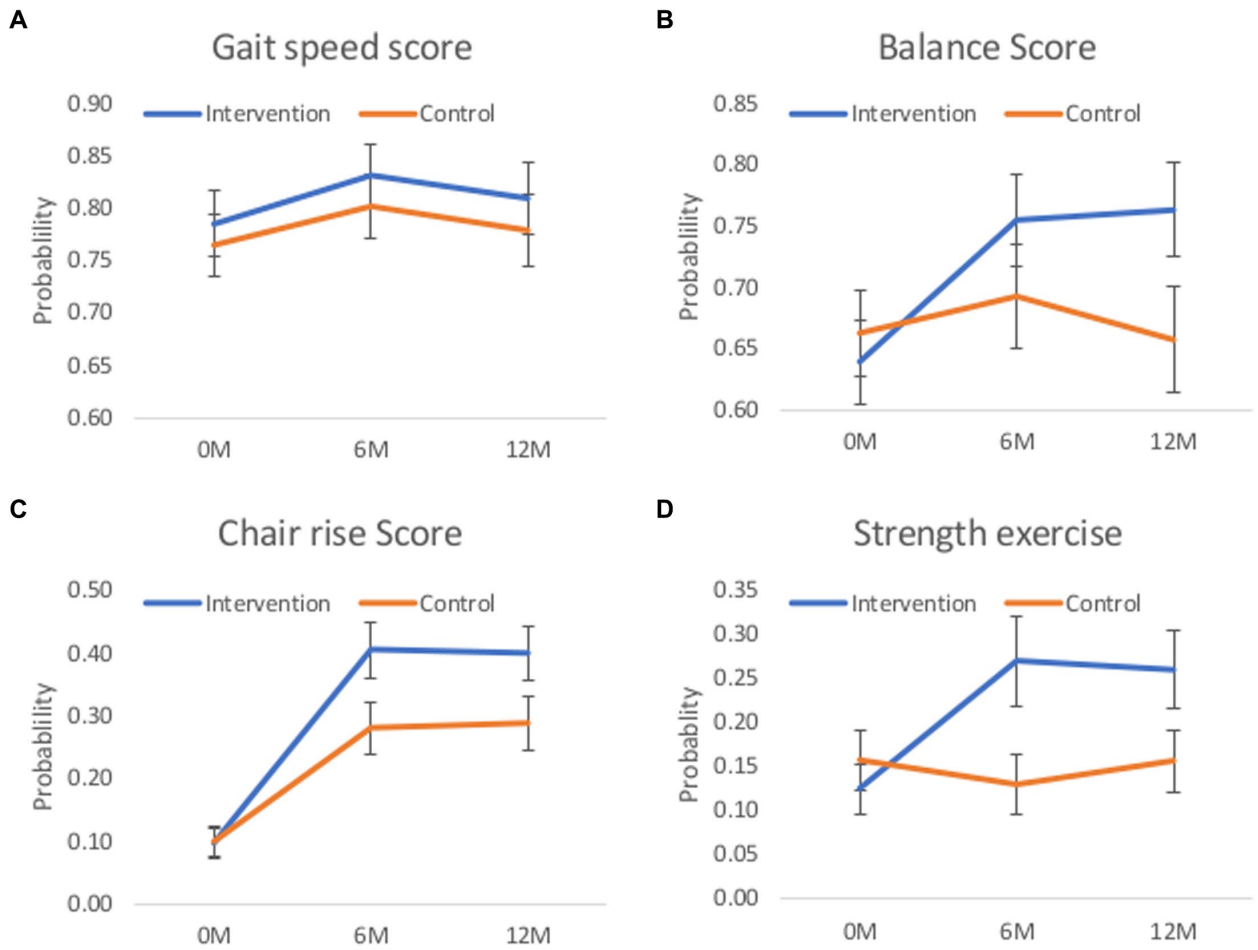
Estimated probability and 95% CI of scoring in the highest two categories (scoring 3 or 4 in the SPPB sub-components or ‘sometimes’ or ‘often’ in strength exercise) in ordinal outcomes. **(A)** gait speed, **(B)** balance, **(C)** chair rise components of the SPPB, and **(D)** the probability of “sometimes” or “often” doing muscle strength and balance exercise in a week. Margins presented at the 6 and 12-month timepoint have been adjusted for any baseline differences between groups in the respective outcome, and the minimisation factors of sex, age, total SPPB score and group exercise site.

Table 4 displays the unadjusted mean (SD) scores for each outcome variable at each time point in the intervention and control groups. The odds ratios (95% CI) and coefficients from the respective mixed logistical or linear models are also displayed for each of the two follow-up time points. Individual component SPPB scores reveal that the exercise intervention improved the odds of demonstrating a greater level of balance (OR = 1.47, 95% CI = 1.07 to 2.02, *p* = 0.017) and lower-limb strength (chair rise scores; OR = 1.96, 95% CI = 1.46 to 2.63, *p* < 0.001) at 6 months. By 12 months, the intervention had heightened the odds of demonstrating superior levels of balance (OR = 1.96, 95% CI = 1.39 to 2.67, p < 0.001) and retained lower-limb strength (OR = 1.88, 95% CI = 1.36 to 2.59, p < 0.001). No significant difference in gait speed scores were observed at any time point between groups. Figure 5 demonstrates the increased probability of participants from the intervention achieving a 3 or 4 (out of 4) in each individual component of the SPPB at 6 and 12 months compared to controls. Specifically, the increased probability of achieving more favourable balance and lower-limb strength profiles (Figures 5B,C). Also, the increased probability of “sometimes” or “often” performing muscle strength/balance exercises in a week (Figure 5D). The analysis of secondary functional outcome measures demonstrate the intervention having favourable effects on perceived mobility and engagement in muscle strengthening exercise at 6 and 12 months (determined using MAT-SF and PASE questionnaire). There was no impact on self-reported falls at either time point, however physical health, as measured by the SF-36, was significantly higher at 6- and 12-months in the intervention group (Table 4).

**Table 4:** Unadjusted mean scores for the outcome variables at each timepoint and the odds ratio (ordinal/binary variables) and coefficients (continuous variables) for outcomes at 6 and 12-, months as determined by the mixed logistical or linear regression models.

Variable		Intervention			Control			Difference			
		*n*	Mean	SD	*n*	Mean	SD	Odds ratio/coefficient	95% CI		*p*
SPPB scores											
Gait speed	0 M	410	3.14	0.82	367	3.06	0.85	–	–	–	–
	6 M	354	3.35	0.85	304	3.25	0.91	1.34	0.95	1.90	0.095
	12 M	346	3.32	0.91	303	3.22	0.92	1.32	0.91	1.90	0.139
Balance	0 M	410	2.90	1.03	367	2.96	1.07	–	–	–	–
	**6 M**	**354**	**3.32**	**1.06**	**305**	**3.10**	**1.12**	**1.47**	**1.07**	**2.02**	**0.017**
	**12 M**	**346**	**3.21**	**1.06**	**303**	**2.94**	**1.17**	**1.92**	**1.39**	**2.67**	**0.000**
Chair rise	0 M	410	1.34	0.86	367	1.32	0.84	–	–	–	–
	**6 M**	**354**	**2.13**	**1.26**	**304**	**1.76**	**1.17**	**1.96**	**1.46**	**2.63**	**0.000**
	**12 M**	**346**	**2.09**	**1.25**	**303**	**1.70**	**1.19**	**1.88**	**1.36**	**2.59**	**0.000**
SPPB continuous											
4MWtime (s)	0 M	406	5.41	1.57	363	5.47	1.76	–	–	–	–
	**6 M**	**343**	**5.04**	**1.57**	**300**	**5.38**	**2.45**	**−0.36**	**−0.61**	**−0.11**	**0.004**
	12 M	343	5.20	2.15	299	5.39	2.51	*−0.15*	*−0.42*	*0.11*	*0.263*
Chair rise time (s)	0 M	333	19.91	8.16	306	19.99	9.73	–	–	–	–
	**6 M**	**297**	**15.13**	**5.26**	**258**	**16.97**	**6.65**	**−1.55**	**−2.57**	**−0.53**	**0.003**
	**12 M**	**299**	**15.29**	**5.08**	**239**	**16.35**	**5.35**	**−1.16**	**−2.01**	**−0.32**	**0.007**
Secondary outcomes											
MAT-SF score	0 M	403	49.06	9.75	357	49.89	8.88	–	–	–	–
	**6 M**	**345**	**51.04**	**10.33**	**297**	**50.26**	**9.68**	**1.35**	**0.30**	**2.40**	**0.012**
	**12 M**	**328**	**51.02**	**10.53**	**292**	**49.53**	**9.82**	**2.13**	**0.97**	**3.30**	**0.000**
SF36-physical	0 M	393	29.70	10.96	352	30.01	10.61	–	–	–	–
	**6 M**	**342**	**32.75**	**11.48**	**293**	**30.61**	**10.88**	**2.09**	**0.78**	**3.40**	**0.002**
	**12 M**	**334**	**31.94**	**11.54**	**293**	**29.77**	**10.89**	**2.57**	**1.26**	**3.87**	**0.000**
Falls	0 M	401	0.69	1.08	359	0.72	1.15	–	–	–	–
	6 M	345	0.61	1.13	302	0.61	1.10	0.84	0.56	1.25	0.392
	12 M	335	0.60	1.09	295	0.61	1.05	0.77	0.52	1.14	0.187
PASE-strength	0 M	401	0.40	0.83	358	0.53	0.96	–	–	–	–
	**6 M**	**347**	**0.92**	**0.97**	**295**	**0.61**	**1.02**	**2.61**	**1.81**	**3.77**	**0.000**
	**12 M**	**333**	**0.89**	**0.92**	**292**	**0.71**	**1.08**	**2.03**	**1.45**	**2.84**	**0.000**

SPPB, short physical performance battery; 4MWT, 4-metre walk time; MAT-SF, Mobility Assessment Tool-short form score; SF36, Short form Health survey 36 item; PASE, Physical activity scale for the older adults. Bold text indicates significant differences (*p* < 0.05) between Intervention and Control participants.

Chair rise time, as a continuous score (Table 4), also improved in the intervention group at the 6 months (OR = −1.55, 95% CI = −2.57 to −0.53, *p* = 0.003) and 12-month time point (OR = −1.16, 95% CI = −2.01 to −0.32, *p* = 0.007), this is despite a reduction from two to one session per week during this period of the intervention. Gait speed, as a continuous score, was favourable at 6-months. No significant interaction between allocation and age, sex, baseline physical function or number of co-morbidities were observed in the analysis of primary outcome data, suggesting the REACT intervention benefited male and female, younger- and older adults, and people of low and moderate physical function similarly.

**Figure 6:**
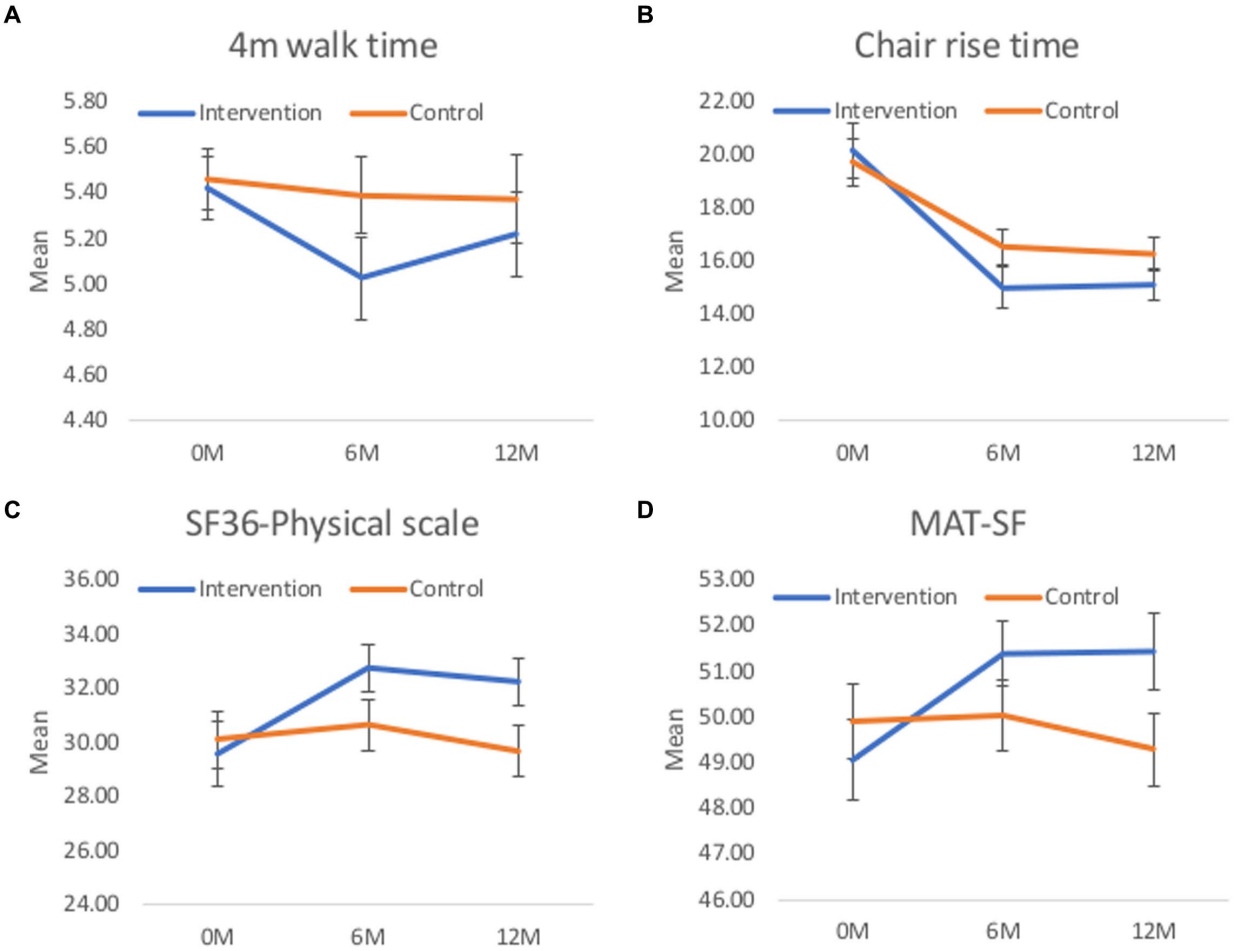
Estimated marginal mean and 95% CI for continuous outcomes. Margins presented at the 6 and 12-month timepoint have been adjusted for any baseline differences between groups in the respective outcome, and the minimisation factors of sex, age, total SPPB score and group exercise site. **(A)** 4m walk time. **(B)** Chair rise time. **(C)** SF36-physical scale. **(D)** MAT-SF.

Figure 6 presents the estimated marginal means from the continuous outcomes for each adjusted mixed multilevel linear models, including (a) 4-metre walking time, (b) chair rise time, (c) SF-36 physical domain, and (d) MAT-SF.

An exploratory post-hoc analysis on the impact of adherence on the component SPPB scores demonstrated that people with high (greater than 75%) adherence to all possible sessions (48 out of 64 exercise sessions) had greater SPPB sub-domain scores than those with low-to-moderate (between 1 and 75%) adherence in both gait speed and chair rise scores, but not balance. This effect was demonstrated at 6 and 12-months (Table 5). Intervention participants who did not attend a single REACT exercise session were not included in this analysis.

**Table 5:** The impact of high (75% or more) vs. low-to-moderate (<75%) adherence on SPPB sub-component outcomes for the REACT exercise intervention participants.

Component		High adherence		Low-to-moderate adherence	
		*n*	Mean (95% CI)^a^	*n*	Mean (95% CI)^a^
Gait speed	**6 M**	**198**	**3.45 (3.35–3.54)**	**123**	**3.27 (3.15–3.39)**
	**12 M**	**182**	**3.45 (3.34–3.56)**	**134**	**3.15 (3.02–3.27)**
Standing balance	6 M	198	3.30 (3.17–3.43)	123	3.23 (3.07–3.39)
	12 M	182	3.33 (3.20–3.48)	134	3.17 (3.01–3.33)
Chair rise	**6 M**	**198**	**2.34 (2.18–2.50)**	**123**	**2.01 (1.81–2.21)**
	**12 M**	**182**	**2.29 (2.12–2.45)**	**134**	**1.87 (1.68–2.07)**

a Estimated means accounting for discrepancies in baseline of dependent variable. Bold text indicates significant differences (*p* < 0.05) between participant’s with high and low adherence rates.

### 3.2. Safety of the exercise intervention

During the 12 month exercise programme there was only one (preventable) serious adverse event that was directly linked to the delivery and management of the exercise sessions for the 410 participants assigned the REACT exercise intervention. This was a fractured hip due to sitting on a faulty chair. One SAE was considered ‘possibly’ related to the REACT programme and this was a transient ischaemic attack (mini-stroke). This SAE occurred outside of the supervised sessions during habitual activity. Based on the small number of SAEs, we consider the REACT programme to be a safe programme to implement for a heterogeneous group of older adults at risk of mobility-related disability.

## 4. Discussion

To our knowledge, the REACT study is the largest translational trial delivered to date targeting long-term changes in lower-limb physical function in older adults with mobility-related disability. Older adults with mobility limitations who received the 12-month exercise intervention demonstrated significant improvements in lower limb function compared to age-matched controls at 6 and 12 months. Physical function appears to peak at 6 months (the most intensive part of the intervention - two sessions per week) and is sustained for an additional 6 months despite reducing to one supervised exercise session per week. Higher intervention effects within individual components of the SPPB were associated with increased attendance to the group-based exercise sessions. Changes in physical function include significantly greater lower-limb muscle strength and balance profiles (assessed using the SPPB test) at 6 and 12 months, which were supported by a higher SF-36 physical component score, MAT-SF score, and self-reported PA and adherence to muscle-strengthening exercise. The subgroup analyses suggested consistency of intervention effects across different subgroups of the population, including sex, people of different ages (over 65 years) and number of comorbidities. These results are consistent with the notion that the exercise intervention increased engagement in muscle strengthening, balance, and endurance activities that mediated the observed effects on physical functioning. Therefore, contrary to the belief that older age comes with an inevitable decline in physical functioning, the REACT study shows that this decline can be slowed or even prevented with modest lifestyle changes.

At the completion of the intervention, significant differences were observed in two of the three components of the SPPB; muscle strength (stand to sit), and balancing tasks, not gait speed. As performing lower-limb strengthening and balance tasks were the cornerstone of the 12 month exercise intervention, the favourable improvements demonstrated in strength and balance are most likely a direct consequence of training specificity and applying the overload principle (progressive intensity of a similar movement pattern performed frequently). No change in gait speed score was a surprising finding given how frequently walking was promoted outside of the REACT sessions (an entire topic was dedicated to PA promotion during the behavioural element of the REACT programme). Whilst the SPPB is a well validated (29) and commonly used measure of physical mobility/function in research performed using older adults (4, 5), based on the verbal instruction provided to participants, it might not be sensitive enough to capture meaningful changes in sub-maximal ambulatory performance (38). Verbal instructions provided during this SPPB task include, “walk at your “usual” walking speed.” Such a verbal instruction does not encourage the participant to demonstrate their peak walking capacity and it may not therefore be sensitive enough to identify changes in ambulatory performance. Between baseline and 12 months, the mean SPPB score for gait speed was “3” (out of 4) for both the intervention and control group. Participant scoring “3” during this task can walk 4 metres between 4.82 and 6.20 s (or between 0.64 m.s^1^ and 0.83 m.s^1^).

Based on this scoring method, no significant differences were reported between intervention and control group at any time point during the intervention. One reason for this might be due to the ‘ceiling effect’. Baseline SPPB gait scores (mean 3.1) were highest of the three sub-domains suggesting the opportunity for group level increases were reduced relative to the lower scores demonstrated in balance (mean 2.9) and chair rise (mean 1.3). However, when expressed as a continual variable (seconds to walk 4 metres), significant difference were demonstrated at 6 months, but not at 12 months. Had the participant been instructed to “safely walk as briskly/fast as you can,” greater opportunity to identify changes in ambulatory function might have been possible. It is therefore likely that the significant differences demonstrated in physical function between the REACT intervention and control groups (as determined by total SPPB score) was underestimated in this study. However, results from accelerometer data (16) suggest PA levels did not change outside of the REACT sessions. Individuals may have compensated for the REACT sessions by reducing other daily activity therefore influencing PA (time spent walking) per week.

There are numerous studies, all using different approaches to multicomponent exercise intervention design, which have elicited favourable changes in mobility-disability and health in community dwelling older adults (4, 5, 39–42). Unfortunately, none of these multicomponent interventions (4, 5, 39–42) performed a within or between group analysis on individual SPPB component scores. It is therefore not possible to identify which component of fitness was driving the favourable changes in physical function reported in these studies. For the advancement of exercise prescription and long term programme design, it would be interesting to know whether the changes in total SPPB scores identified in these (and future) studies are primarily driven by strength and balance scores. Also, given the standardised instructions provided to assessors, whether these studies also propose a potential ceiling effect with the gait speed component of the SPPB, as demonstrated in the REACT study.

Despite being a safe exercise intervention that demonstrates improvements in physical function, falls and fall-related injuries were not found to be significantly reduced by the REACT intervention. This finding is in line with both the LIFE (4) and SPRINTT (5) PA interventions and suggests that the REACT intervention alone may not be adequate for preventing falls in individuals with mobility limitations. However, a limitation of each of these studies is the reliability associated with retrospectively self-reporting the incidence of falling. It is important to note that REACT was not designed to be a falls prevention intervention. No assessment and/or modification of the home/local environment or monitoring of any drug withdrawal were performed during the REACT intervention, which are typical of successful falls prevention strategies (43). The multimodal holistic nature of the REACT exercise intervention (with a strong behavioural maintenance element) aimed to reduce mobility-related disability by improving overall physical function, increasing independence, social networks and making everyday tasks easier to perform. It is important to note that no differences in the number of SAEs were reported between the two groups in the main study (16).

Resistance training is commonly performed using specialist equipment (i.e., free weights, resistance machines etc.) and international organisations, such as the American College of Sport Medicine [ACSM] (44) and NSCA (10), often emphasise these approaches in their guidelines. The exercise prescription recommendations provided by these organisations are often time consuming, require heavy loads for resistance and expensive infrastructure to deliver. The REACT programme provides a safe, pragmatic and cost effective method of reducing mobility-related disability in older adults within the community setting with as little as one exercise session per week (a fairly low level of commitment) requiring little in the way of expensive resources. Designed to meet both national and international PA guidelines (6, 8), the multimodal exercise intervention is unique in its management of older adults offering personalised exercise prescription that can be regressed and progressed to meet the functional and energy needs of the individuals over a 12 month duration. Providing a large selection of exercises reduces monotony and facilitates a logical progression of physical function through graduated exposure to higher intensity or functionally demanding exercise selection (sitting to standing with support to standing independently to multidirectional movements and jumping). These findings provide important evidence informing future national/international PA and exercise guidelines for multimodal exercise for older adults (6, 8, 10). The exercise protocol (Supplementary File S1) is clearly described making the REACT intervention reproducible at scale across the United Kingdom.

### 4.1. Recommendations for future long-term exercise interventions

Pragmatic research trials are designed to assess the effectiveness of an intervention as it would be delivered in the real world, rather than under highly controlled conditions. More pragmatic research trials, embedded within a community setting, are needed to advance our understanding of the benefits of PA and exercise in public health. We support the recent call for action for better reporting of exercise interventions across a range of health conditions (45). Previous research trials describe their long term exercise programmes (across all age groups) poorly. Transparency surrounding the methods adopted when performing a needs analysis is essential for the future advancement of exercise knowledge and dissemination of research findings.

When designing the 12 month REACT exercise intervention the basic principles of training were followed (the principles of specificity, overload and variation) (46). Progressive exercises were modified where necessary to meet the functional needs of the population (i.e., if a pre-existing medical condition or MSK pain restricts specific movements). All medium to long-term exercise programmes should be flexible and varied enough to offer progression and regression of exercise intensity/difficulty when appropriate. Within the main REACT study findings, increases in adherence to the exercise programme were associated with superior global SPPB scores (16). When analysed according to each individual SPPB sub-component score, the improvements demonstrated in balance were not dependent on high adherence to the exercise intervention (Table 5). However, high adherence (>75%, or attending >48 out of 64 exercise sessions) was required to elicit improvements in the muscle strength and gait speed at 6 and 12 months.

Clinically meaningful changes in physical function were demonstrated using bodyweight exercises at moderate intensities with minimal equipment. The strength and balance components of the supervised exercise sessions adopted a 3 × 2 superset block design (making session delivery more time efficient) in combination with easy to understand methods of monitoring exercise intensity (RPE and RIR) which are helpful when managing the expected fluctuations in energy levels or fatigue documented in this type of population (further details on exercise protocol can be found in Supplementary File S1). REACT represents an effective and cost effective methodological approach to exercise prescription and programme design. To further our understanding of how best to integrate exercise interventions (at low cost) for older adults at risk of mobility disability, the evaluation of implementation of the REACT programme at a national level is required. Also, the evaluation and health economic evaluation of other exercise programme methodologies delivered within a community setting are warranted.

The total SPPB score offers researchers a useful global measure of physical function, but hides the underlying mechanisms underpinning any functional changes demonstrated as a result of engaging in any form of exercise intervention. This study has highlighted potential limitations within the interpretation of the gait speed component of this commonly used outcome measure. Researchers/clinicians are encouraged to be mindful of how they report and interpret global physical function SPPB scores given this new insight. Breaking down the SPPB into its component parts and/or combining the SPPB with other objective markers of physical function may prove useful when trying the better understand the mechanisms underpinning functional changes to exercise intervention for older adults (38).

### 4.2. Considerations prior to any future implementation of the REACT exercise intervention

REACT provides local, regional and national service providers an effective (16) and cost effective (15) solution to reduce the risk of mobility disability (inclusive of age, sex, number of comorbidities and SPPB score between 4 and 9). REACT could be offered as an intervention before ambulatory-based independence become harder to achieve and secondary heath concerns become more challenging to treat and/or manage (i.e., prior to the deleterious effects of frailty and its associated healthcare costs take effect). Alternatively, it might also be possible and synergistic to integrate the REACT intervention with existing mobility-related prevention and rehabilitation services available within the community. Delivery could be in partnership with other rehabilitation professionals across healthcare and community settings as happens within ‘falls prevention’ exercise delivery. This approach could support continued progression and long-term engagement but would require barriers to participation such as accessibility to be understood and addressed (47).

Being successfully delivered by “level 3” exercise practitioner’s enables care providers and policy makers a greater number of qualified exercise professionals (i.e., gym instructors and personal trainers) to recruit and manage exercise sessions within their region. This makes delivering such programmes more cost effective and sustainable compared to relying upon healthcare roles such as physiotherapists or clinical exercise physiologists. The practise of combining health economic evaluations alongside exercise interventions should be widely encouraged by the research community, policy makers and service providers. To our knowledge, REACT is the first exercise programme delivered alongside a health economic evaluation for older adults at risk of mobility disability. The cost of delivering the 12 month REACT programme was estimated to be £622 per participant (15). This cost could be more than offset by reductions in resource use of social services and the National Health Service (NHS), in particular relating to secondary care, generating net cost savings and improved health-related quality of life.

To facilitate additional strength adaptations over the longer-term and optimise adaptations in bone mineral density, access to provision or equipment that enables heavier load resistance training in accordance with NSCA recommendations (10) (i.e., access to local gym with fixed and free-weight resistance training equipment) may be warranted. Helpful resources relating to resistance exercise prescription and the proposed benefits of higher intensity loading paradigms for the benefits of public health are available elsewhere (48–50). However, for the majority of individuals at risk of mobility disability, exercise practitioners should not view a lack of equipment as a barrier to effective exercise delivery or an insufficient stimulus to elicit favourable adaptations to training.

## 5. Conclusion

The REACT programme provides important evidence supporting WHO, United States, and United Kingdom PA recommendations for multimodal exercise for adults older than 65 years at risk of mobility disability (6, 8, 9). The 12 month REACT programme demonstrated improvements in physical function regardless of age, sex, baseline function and number of co-morbidities. This was achieved employing level 3 gym instructors without the provision of expensive cardiovascular or resistance training equipment, with as little as one multimodal exercise session per week demonstrating a benefit. Improvements in total SPPB scores were driven by changes in strength and balance, not gait speed. Upon reviewing the guidelines to SPPB administrators, we propose that the instructions provided to participants during the gait speed component of the SPPB are inappropriate to determine ‘peak’ ambulatory performance. Instead, they provide a useful determinant of ‘habitual’ ambulatory performance. Participants were therefore unable to demonstrate their ambulatory ‘capacity’ using the SPPB, suggesting changes in physical function may have been underestimated following the REACT intervention. We consider the SPPB to be a useful measure of global physical function. However, a 4 metre gait speed assessment (as used in the SPPB) might not be sensitive enough to characterise peak ambulatory performance. The inclusion of a longer duration ambulatory-based outcome measure (such as the six-minute walk test) may prove to be a beneficial adjunct when establishing ‘peak ambulatory capacity’ in older adult populations.

More transparent and detailed reporting of exercise interventions across a range of health conditions is essential for the future advancement of exercise knowledge and dissemination of research findings. Exercise programmes for older adults at high risk of mobility disability should be flexible and varied enough to offer progression and regression of exercise intensity/difficulty based on the energy status of the individual. These analyses will aid the development of future recommendations of good practise on how best to support older adults to maintain an active lifestyle and quality of life. Exercise-based interventions (with detailed exercise protocols provided) that utilise a similar methodological approach for frail older adults (i.e., SPPB score less than 4 (not captured by REACT)) are warranted. There is now a requirement for implementation studies evaluating the impact of REACT in a wide community role out.

## Data availability statement

The datasets presented in this study can be found in online repositories. The names of the repository/repositories and accession number(s) can be found at: All source data used in this study are publicly available. The trial dataset can be accessed by contacting AS (A.Stathi@bham.ac.uk). All project documentation is available at https://www.fundingawards.nihr.ac.uk/award/13/164/51.

## Ethics statement

The studies involving human participants were reviewed and approved by National Health Service (NHS) South East Coast – Surrey Research Ethics Committee (15/LO/2082). The patients/participants provided their written informed consent to participate in this study.

## Author contributions

AS led the study as chief investigator. AS, CG, JT, and KF obtained funding for the research. AS, PL, CG, JT, JW, JG, and KF contributed to the design of the main REACT Study. JW led the coordination of the REACT study as the trial manager. PL, MW, CG, JW, and AS conceptualised the study design of this article. PL designed the REACT exercise protocol and wrote the original draught with critical input from all other authors. JW, JK, JB, and SM were involved in the collection and processing of data. MW performed formal data analysis. All authors contributed to the article and approved the submitted version.

## Funding

This work was supported by the NIHR Public Health Research Programme (13/164/51).

## Conflict of interest

The authors declare that the research was conducted in the absence of any commercial or financial relationships that could be construed as a potential conflict of interest.

## Publisher’s note

All claims expressed in this article are solely those of the authors and do not necessarily represent those of their affiliated organizations, or those of the publisher, the editors and the reviewers. Any product that may be evaluated in this article, or claim that may be made by its manufacturer, is not guaranteed or endorsed by the publisher.

## Author disclaimer

This publication presents independent research funded by the National Institute for Health and Care Research (NIHR). The views and opinions expressed by authors in this publication are those of the authors and do not necessarily reflect those of the NHS, the NIHR, the PHR programme or the Department of Health and Social Care.
