# Supplementary material for: The REtirement in ACTion exercise programme and its effects on elements of long term functionality in older adults
Source: Front Public Health. 2023 Jul 28;11:1151035. doi: 10.3389/fpubh.2023.1151035 (PMC10420051; doi:10.3389/fpubh.2023.1151035)
Supplement: Supplementary file 1 [file Table_1.DOCX]

**Supplementary File 1:** Protocol for The Exercise Component of The Retirement in ACTion (REACT) Study: A Multi-Centre Randomised Controlled Trial

**Created by:** Dr Peter Ladlow

This document describes the background to the exercise component of the REACT study programme. For a detailed description of the entire protocol, readers are encouraged to review the following protocol paper:(1)

Stathi A, et al. A community-based physical activity intervention to prevent mobility-related disability for retired older people (REtirement in ACTion (REACT)): study protocol for a randomised controlled trial. *Trials*, 2018, 19:228

For a description of 1) the main study findings,(2) 2) cost effectiveness of the trial(3) and 3) fidelity of the accompanying behavioural change component of the REACT study,(4) readers are encouraged to read the following articles:

Stathi A, Greaves CJ, Thompson JL, et al. Effect of a physical activity and behaviour maintenance programme on functional mobility decline in older adults: the REACT (Retirement in Action) randomised controlled trial. *Lancet Public Health*, 2022, 7, e316-326.

Snowsill TM, Stathi A, Green C, et al. Cost-effectiveness of a physical activity and behaviour maintenance programme on functional mobility decline in older adults: an economic evaluation of the REACT (Retirement in Action) trial. *Lancet Public Health* 2022; 7, e327-334.

Cross R, Greaves CJ, Withall J, et al. Delivery fidelity of the REACT (REtirement in ACTion) physical activity and behaviour maintenance intervention for community dwelling older people with mobility limitations. BMJ Public Health3;22(1):1112.

Contents

[1.1 REACT Study: Background 4](#_Toc107823010)

[2.1 Existing National and International guidelines on PA and Exercise for older adults 4](#_Toc107823011)

[2.1.1 2019 UK Chief Medical Officers’ Physical Activity Guidelines(6) 4](#_Toc107823012)

[2.1.2 US Department of Health and Human Services (HHS) Office of Disease Prevention and Health Promotion 2018 Physical Activity Guidelines for Americans (2^nd^ Edition)(7) 5](#_Toc107823013)

[2.1.3 Resistance Training for Older Adults: 2019 Position Statement from the National Strength and Conditioning Association (NSCA) (8) 5](#_Toc107823014)

[2.1.4 An important note on the NSCA general recommendations and its implications for the REACT exercise intervention 6](#_Toc107823015)

[3.1 The REACT Study: Generic Structure (Week 1 to week 52) 6](#_Toc107823016)

[3.1.1 Start-up (adoption: weeks 1–8) 6](#_Toc107823017)

[3.1.2 Build-up (adoption: weeks 9–24) 6](#_Toc107823018)

[3.1.3 Taking charge (maintenance: weeks 25 to 52) 6](#_Toc107823019)

[3.2 The REACT Study: Two-day Training Course Delivered to *the Exercise Leaders* 7](#_Toc107823020)

[3.3 The REACT Study: Infrastructure and Resources Available During Supervised Exercise Sessions 7](#_Toc107823021)

[3.4 The REACT Study: Expected Functional Status of Participant’s at Baseline 7](#_Toc107823022)

[3.4.1 Eligibility criteria 7](#_Toc107823023)

[3.4.2 Implications of eligibility criteria on baseline functional status and introductory exercise sessions 8](#_Toc107823024)

[3.5 The REACT Study: Functional Outcome Measures 9](#_Toc107823025)

[3.5.1 Primary functional outcome measure 9](#_Toc107823026)

[The primary outcome measure used in the REACT study is the short physical performance battery (SPPB. This objective outcome measure consists of using a composite score of three functional assessments: 9](#_Toc107823027)

[3.5.2 Secondary functional outcomes measures: 9](#_Toc107823028)

[3.6 The REACT Study: Generic Framework for 12 month of progressive exercise delivery 10](#_Toc107823029)

[3.6.1 Progressive Functional Continuum 10](#_Toc107823030)

[3.6.1a Inclusion of explosive movements into the 12 month functional continuum 10](#_Toc107823031)

[3.6.1b Use of upper limb exercises 11](#_Toc107823032)

[3.6.2 General principles of exercise progression 13](#_Toc107823033)

[3.6.4 Choices and Constraints: Transitioning through the functional continuum as time and physical function progress 15](#_Toc107823034)

[3.6.5 Strategies to promote PA outside of the structured exercise sessions 16](#_Toc107823035)

[3.7 The REACT Study: Personalised Exercise Program 19](#_Toc107823036)

[3.7.1 Determining exercise selection for the first exercise session 19](#_Toc107823037)

[3.7.2 Structure of each REACT exercise session 19](#_Toc107823038)

[3.7.3 Selecting and monitoring exercise intensity 21](#_Toc107823039)

[3.7.4 Home-based exercise sessions 21](#_Toc107823040)

[3.8 The REACT Study: A summary of how the exercise protocol meets national / international PA and exercise guidelines for older adults 21](#_Toc107823041)

[4.1 Potential implications of findings 22](#_Toc107823042)

[5.1 References 22](#_Toc107823043)

# 1.1 REACT Study: Background

There have been few attempts to develop, and rigorously evaluate, feasible models of PA promotion for older people in community settings. The REtirement in ACTion (REACT) study represents the first large-scale, pragmatic, community-based trial in the UK to target the non-disabled but high-risk segment of the older population with an intervention to reduce mobility-related disability. People in this category are still physically capable of engaging in a progressive exercise programme and have potential for prevention of further physical decline. An exercise programme that can successfully engage them in sufficient activity to improve strength, aerobic capacity, coordination and balance would have a major impact on their prospects for sustained health and independence.(5) The motivation behind the REACT study was the findings from the landmark study of PA promotion in older adults, the ‘Lifestyle Interventions and Independence for Elders’ (LIFE) delivered in the United States.(5) LIFE was a multi-centre randomised controlled trial comparing the effects of a PA programme with a successful ageing educational programme in more than 1600 functionally compromised older adults. After an average 2.6 years follow-up, the intervention reduced the risk of developing major mobility disability (defined as the inability to complete a 400 metres walk test within 15 minutes) by 18% and persistent mobility disability by 28%, relative to the control group. The intervention group maintained an increase of 40 minutes per week in objectively assessed lifestyle-intensity activity (≥ 760 counts per minute compared with the control group at 24 months of follow-up). These estimates are likely to be conservative as the study utilised an active control group which received a substantial health education/lifestyle intervention including weekly workshops for 6 months and monthly sessions for a further 18 months.

# 2.1 Existing National and International guidelines on PA and Exercise for older adults

## 2.1.1 2019 UK Chief Medical Officers’ Physical Activity Guidelines(6)

Older adults should participate in daily PA to gain health benefits. Some PA is better than none: even light activity brings some health benefits compared to being sedentary.

Older adults should break up prolonged periods of being sedentary with light activity when physically possible, or at least with standing, as this has distinct health benefits for older people.

Older adults should maintain or improve their physical function by undertaking activities aimed at improving or maintaining muscle strength, balance and flexibility on at least two days a week. These could be combined with sessions involving moderate aerobic activity or could be additional sessions aimed specifically at these components of fitness.

Each week older adults should aim to accumulate at least 150 minutes of moderate intensity aerobic activity, building up gradually from current levels.

Those who are already regularly active can achieve these benefits through 75 minutes of vigorous intensity activity, or a combination of moderate and vigorous activity, to achieve greater benefits. Weight-bearing activities which create an impact through the body help to maintain bone health.

Older adults should break up prolonged periods of being sedentary with light activity when physically possible, or at least with standing, as this has distinct health benefits for older people.

## 2.1.2 US Department of Health and Human Services (HHS) Office of Disease Prevention and Health Promotion 2018 Physical Activity Guidelines for Americans (2^nd^ Edition)(7)

Older adults should move more and sit less throughout the day. Some PA is better than none.

Older adults should do at least 150 minutes to 300 minutes a week of moderate-intensity, or 75 minutes to 150 minutes a week of vigorous-intensity aerobic PA, or an equivalent combination of moderate and vigorous-intensity aerobic activity. Preferably, aerobic activity should be spread throughout the week.

Adults should also do muscle-strengthening activities of moderate or greater intensity that involve all major muscle groups on 2 or more days a week.

As part of their weekly PA, older adults should do multicomponent PA that includes balance training as well as aerobic and muscle-strengthening activities. Older adults should determine their level of effort for PA relative to their level of fitness.

When older adults with chronic conditions or disabilities are not able to meet the above key guidelines, they should engage in regular PA according to their abilities and should avoid inactivity.

## *2.1.3 Resistance Training for Older Adults: 2019 Position Statement from the National Strength and Conditioning Association (NSCA)* (8)

Resistance training programs for older adults should follow the principles of individualisation, periodisation, and progression. They should ideally be monitored and designed to match the unique physical, psychological, and medical challenges of the individual.

Multicomponent exercise intervention programs that consist of resistance training, gait retraining, and balance training seems to be the best strategy for improving gait, balance, and strength, as well as reducing the rate of falls in older adults and consequently maintaining their functional capacity during aging. A summary of the NSCA’s general recommendations at provided in table 1.

Table 1: NSCA’s general recommendations for resistance training in healthy older adults(8)

| **Programme variable** | **Recommendation** |
| --- | --- |
| Sets | 1 to 3 per exercise per muscle group |
| Repetitions | 8 to 12 or 10 to 15 |
| Intensity | 70 to 85% of 1repetition maximum (1RM) |
| Exercise selection | 8 to 10 different exercises |
| Modality | Free weight or machine-based exercises |
| Frequency | 2 to 3 days per week, per muscle group |
| Power/explosive training | 40 to 60% of 1RM |
| Functional movements | Exercises to mimic tasks of daily living |

## 2.1.4 An important note on the NSCA general recommendations and its implications for the REACT exercise intervention

We recognise the importance of position stands/consensus statements on resistance training guidelines like those provided by the NSCA (table 1). However a significant proportion of their recommendations rely on expensive infrastructure and resources (exercise equipment) that simply aren’t available for the majority of local councils/charities who are trying to establish cost effective strategies of improving the long-term health of older adults in their communities.

To ensure the REACT intervention is reproducible at large scale within the UK (i.e., sustainable) will require a less prescriptive approach to resistance training delivery that relies on little to no equipment, which implies significantly reduced training load (intensity). Importantly previous research has demonstrated that when one’s own body weight is used for resistance and in which activities of daily living are simulated (for example, body weight squat) can improve indices of physical function in older adults to a similar extent as conventional resistance training methods that require external loads.(9) Further detail relating the generic framework and individualisation of weekly programmes are discussed in sections 3.6 and 3.7.

# 3.1 The REACT Study: Generic Structure (Week 1 to week 52)

## 3.1.1 Start-up (adoption: weeks 1–8)

*T*he purpose of this phase is to stimulate initial increases in PA and fitness, to reduce any anxieties or concerns about exercise, and to build confidence and a sense of attachment to the programme.

- Each participant will receive a 45-minute individualised, face-to-face introductory session which will be used to personalise the programme for starting levels and progression.
- Two 60-minute PA sessions per week, plus 15–20 minute of social time, will then be delivered by the REACT trainer.

## 3.1.2 Build-up (adoption: weeks 9–24)

A 45-minute interactive educational/social session run by the REACT trainers will be added at the end of one of the two weekly sessions. These sessions will use evidence-based, person centred behaviour-change strategies to build intrinsic motivation and self-efficacy. They will be designed to maximise enjoyment, social interaction and group identity.(10) Behavioural management will focus on self-regulation using goal setting, self-monitoring, reviewing of goals and problem-solving.(11, 12) A key focus will be on exploring and planning transition to more lifestyle-based activities.

- Pedometers will be introduced during these sessions to support the participant in the transition to the maintenance phase.
- After week 12, the exercise session frequency is reduced to once per week but with an expectation that participants find an hour per week to exercise at home, in the neighbourhood or at a PA session in their local community.

## 3.1.3 Taking charge (maintenance: weeks 25 to 52)

The maintenance stage will focus further on home and neighbourhood-based activities while continuing with a weekly centre-based PA session followed by a short social session.

- Supervised exercise frequency of once per week remains
- Participants will enact action plans for PA outside of the REACT programme that were made during the transition phase and will be supported through group social/education meetings once a month.
- Encourage groups to self-organise their own social interaction beyond the scope of the study and to consider doing activities together.
- Participants will be informed about local opportunities for PA in their community.

# 3.2 The REACT Study: Two-day Training Course Delivered to *the Exercise Leaders*

Two days of face-to-face intervention delivery training to session leaders was provided, including detailed session plans to ensure consistency in, and fidelity to, programme delivery based on the REACT exercise intervention guide. Training delivered at each regional location and consisted of both theoretical and practical examples relating to the integration of exercise and behaviour change strategies. Training as delivered to exercise professional with a minimum of a level 3 NVQ exercise instructor qualification (REPS level 3) with experience of working with older adult populations.

Training relating to the exercise component focussed on ensuring the exercise leaders are familiar with the standardised exercise intervention guide. This manual provides a rationale behind the exercise intervention, the framework for exercise delivery, exercise session templates and a catalogue of over 100 exercises and games (ranging in functional and cognitive difficulty) that the exercise leaders can incorporate into their weekly exercise programme. Additional details relating to the content within this intervention guide is provided below.

# 3.3 The REACT Study: Infrastructure and Resources Available During Supervised Exercise Sessions

The REACT study will be conducted at three UK sites – Bath/Bristol, Devon and Birmingham. This would allow recruitment of a socio-economically and ethnically diverse sample including participants from urban, rural and semi-rural locations. To ensure the REACT study could be delivered at cost and at a large scale, group exercise sessions were delivered in local community/leisure centres during off-peak hours. Sessions will be organised as group activities with up to 15 participants per group. No access to resistance machines, free-weights or cardiovascular machines would be available during supervised sessions to facilitate improvements in muscle strength, muscle hypertrophy or aerobic capacity. However, each centre location ensured enough chairs were provided for participants to use if/when required and elastic therabands and ankle weights were provided by the REACT research team to be used a method of resistance.

# 3.4 The REACT Study: Expected Functional Status of Participant’s at Baseline

## 3.4.1 Eligibility criteria

The eligibility criteria is intended to identify 768 sedentary, community-dwelling, older people aged 65 years and over with functional limitations (i.e. who are at risk of major mobility limitations), but who are still ambulatory, i.e. they can still walk. This will be measured using a battery of objective physical function tests (Short Physical Performance Battery (SPPB)) to assess balance, walking speed and the ability to change from a sitting to a standing position. This generates a physical function score from 0 to 12. Older adults with scores of 4–9 (inclusive) out of 12 were eligible to take part in REACT. This is based on data showing that older adults with SPPB scores of 9 or less have substantially higher risk of major mobility disability 3 years later compared with those with a score of 12.(5, 13)

The exclusion criteria for the REACT study is provided in table 2, a comprehensive eligibility criteria is provided in the main protocol paper.(1)

**Table 2:** REACT study exclusion criteria, taken from Afroditi et al.(1)

| 1. Existing major mobility limitation (defined as SPPB of 3 or less, or unable to complete the 4-m walk component of SPPB)  2. Living in residential or nursing care  3. Inability to attend the REACT physical activity sessions as scheduled  4. A documented or patient-reported medical condition that would preclude participation, including:  • Arthritis so severe it would prevent participation in physical activity  • Parkinson’s disease or diagnosed dementia  • Any terminal illness  • Lung disease requiring use of orally administered corticosteroids or supplemental oxygen  • Severe kidney disease requiring dialysis  • Severe heart disease that would prevent participation in physical activity (e.g. chest pain when walking 100 or 200 yards or up a flight of stairs)  • Implanted cardiac defibrillator  • Cardiac arrest which required resuscitation  • Severe uncontrolled psychiatric illness  • Currently receiving radiation therapy or chemotherapy treatment for cancer  • Awaiting knee or hip surgery  • Major heart surgery (including valve replacement or bypass surgery) in the last 6 months  • Unstable heart condition (e.g. uncontrolled arrhythmia, angina, heart failure or hypertension)  • Spinal surgery in the last 6 months  • Any other clinical condition that the person’s GP or clinician considers would make them unsuitable for participation in a physical activity rehabilitation programme to prevent decline of lower-limb functioning  **Temporary exclusion criteria:**  5. Heart attack (or myocardial infarction), stroke, spinal surgery, hip fracture, hip or knee replacement within the previous 6 months  6. Currently receiving physical therapy on legs or enrolled in another physical activity research or intervention study  7. Unable to walk across a room without the help of another person, living in residential care, awaiting hip or knee surgery, or receiving radiation therapy or chemotherapy, along with people who had had recent heart or spinal surgery or had an illness that would prevent participation, such as those with severe arthritis, diagnosed moderate-to-severe dementia, severe kidney disease, unstable heart disease, and severe psychiatric illness. |
| --- |

## 3.4.2 Implications of eligibility criteria on baseline functional status and introductory exercise sessions

The key exclusion criteria detailed in Table 2 include medical conditions that would preclude participation in gentle PA, living in residential or nursing care, and an inability to attend scheduled REACT PA sessions. However, due to the broadness of the inclusion criteria, the exercise protocol must be inclusive towards a variety of existing chronic health conditions, including; hypertension, stroke, cardiovascular disease, diabetes mellitus, COPD, asthma, osteoarthritis, osteoporosis and early onset dementia. All of which have contraindications relating to exercise prescription.

The eligibility criteria provides no restrictions on body composition, meaning participants could be under-weight or clinically obese. In some participants (for example, those with rheumatoid or osteoarthritis), persistent joint pain may be a primary limiting factor to adherence and progress which presents its own unique challenges regarding modifying exercise prescription to meet the functional needs of the individual. Participant age is likely to be broad (between 65 and 100 years); therefore the effects of ‘normal’ biological aging (i.e., sarcopenia) may be more pronounced in some individuals than others making the speed of neuromuscular adaptations to exercise training even more heterogeneous. There is an increased likelihood of a history of falls in this population and many of these individual are likely to lack confidence when engaging in PA or structure exercise due to their perceived re-injury risk. Any underlying anxiety must be managed carefully to ensure trust between the exercise leader and participant. Key to this is selecting an appropriate level of exercise intensity during the early sessions (see section 3.7.1). Communication between participant and exercise leader may also be challenging due to English not be everyone’s first language, some individuals may have sensory deficits (vision and hearing), and some may have moderately impaired cognitive function or learning difficulties.

**Exercise prescription will be driven by the functional requirements of the individual and not on the diagnosis of disease.**

# 3.5 The REACT Study: Functional Outcome Measures

Outcome measures relating to physical function will be collected at four time points; baseline, 6 months (midway through intervention), 12 months (end of intervention) and 24 months follow-up. For a comprehensive list of all REACT study outcome measures please read Stathi et al.(1)

## 3.5.1 Primary functional outcome measure

## The primary outcome measure used in the REACT study is the short physical performance battery (SPPB. This objective outcome measure consists of using a composite score of three functional assessments:

1. Balance,
2. Walking speed,
3. The ability to change from a sitting to a standing position.

## 3.5.2 Secondary functional outcomes measures:

Objective:

- Accelerometer assessed PA
- Hand-grip (dynamometer) strength

Subjective:

- Muscle Strengthening Exercise Adherence Questionnaire
- Subjective physical activity (PASE questionnaire-10 item)
- Physical component of the SF-36 questionnaire
- Mobility Assessment Tool-Short Form (MAT-SF)
- Falls Inventory

# 3.6 The REACT Study: Generic Framework for 12 month of progressive exercise delivery

There is a lack of transparency from previous research on how exercise sessions were successfully or unsuccessfully delivered. There is also an absence of guideline on how to progress exercises during long term PA interventions with older adults. Periodisation, or the phased manipulation of training variables across a given time period in order to best prepare an individual to achieve optimal human performance,(14) has long been a part of the vocabulary of strength and conditioning coaches, but only in relation to planning performance programmes for athlete populations. Whilst there is strong evidence to support progressive periodised exercise programming to develop muscle strength, hypertrophy and endurance in young adults, the application of different progressive exercise training models to support continual improvements in health, function and well-being of elderly adults is seriously lacking. However, multimodal exercise prescription (or concurrent training methods) in older adults appears to be the most effective strategy to counteract declines in muscle mass, strength, cardiorespiratory fitness, neuromuscular function, and functional capacity.(15)

The objective of the REACT exercise protocol is to primarily develop lower-limb function. The rationale for this is because age-related reductions in muscular strength and muscle mass tends to be more pronounced in the lower versus upper limbs.(16) Lower-limb weakness can compromise the ability to perform activities of daily living, leading to a loss of functional independence and increased risk of falls resulting in injury.(17) A reduced ability to walk can predict future disability and older adults who have difficulty walking subsequently have a greater risk for mortality.(18)

## 3.6.1 Progressive Functional Continuum

The progressive functional continuum (figure 1) forms the basis of exercise progression for all strengthening and balance-based exercises over the 12 month REACT intervention. The continuum provides a pragmatic approach to exercise selection and progression that the exercise leaders can incorporate into their session plan. Each of the 5 categories has between 10 and 30 exercises to choose from.

It is important to note that the ‘progressive functional continuum’ does not classify all exercises labelled ‘multidirectional / explosive movement patterns’ (blue) to be of greater intensity/difficulty than all the ‘standing without support’ exercises (orange and red). Nor that all ‘single leg exercises’ (red) are of greater intensity/difficulty than all ‘standing with support’ (purple). When the exercise leader is seeking a new exercise for their next session, a progression or sensible alternative mobility, sensorimotor or strength challenge compared to the current choice of exercise is likely offered as the continuum progresses to the right (see figures in section 3.6.2).

### 3.6.1a Inclusion of explosive movements into the 12 month functional continuum

The inclusion of explosive movements within the ‘functional continuum’ is necessary because explosive strength (or power) decreases at a higher rate with ageing, and is a strong predictor of functional disability in the elderly.(19) Previous research has demonstrated that resistance training programs that aim to improve muscle power are more effective at improving lower-body function than more traditional slower contraction speed exercise programs.(20-22) Explosive movements are not permitted in the REACT exercise programme until after the end of the ‘start-up’ phase (week 8 onwards). See ‘choices and constraints’ in section 3.6.4 for further details

### 3.6.1b Use of upper limb exercises

Whilst not incorporated into the functional continuum, the REACT exercise guide also includes 20 upper-limb exercises that use elastic bands and drink bottles as the primary mode of resistance. Upper limb muscle strength is important for performing activities of daily living such as bathing, dressing, feeding, cleaning, cooking, and others. Therefore, maintaining upper body strength is critical for maintaining functional capacity during aging. Specific movements such as pushing, pulling, lifting, holding, trunk flexion, trunk rotation and stabilization are incorporated into the list of available exercises for REACT leaders to select. How upper limb exercises are integrated into the each individual’s weekly programmes is detailed in section 3.7.2.


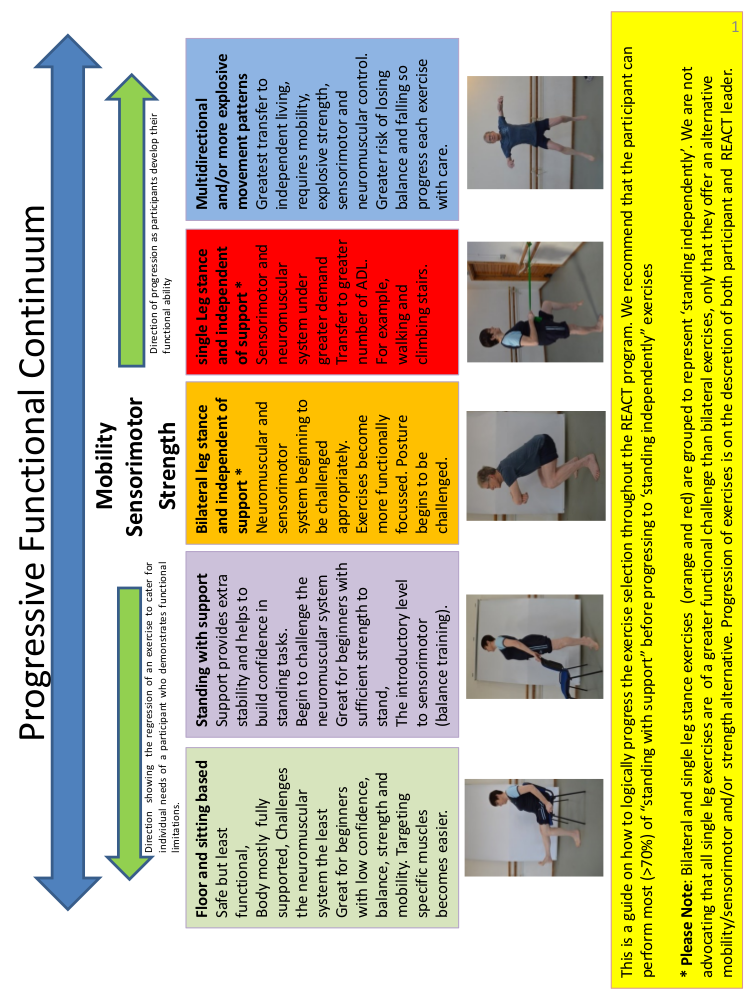


**Figure 1:** REACT Study Progressive Functional Continuum

## 3.6.2 General principles of exercise progression

Clear clinical reasoning relating to appropriate progression and regression of exercise is essential to achieve positive functional outcomes.(23) The ideal progression model to use with an older adult is one in which there is a smooth increase in loading intensity that optimizes strength gains while preserving interest levels and enjoyment.(24)

Commonly used methods to determine the appropriateness of exercise prescription and progression (for example, heart rate monitoring predictions of one-repetition maximum tests) were not available. Organisations such as NSCA encourage strengthening based training for older adults, however their recommendations heavily emphasise the use of heavy load resistance training through the use of free-weights or fixed machines, equipment not available within our REACT sessions. Therefore, the REACT exercises will be primarily based on callisthenics (body weight exercises) of varying functional difficulties.

The model used for progressing exercise prescription within the REACT study is provided in figure 2. This model was inspired by *Blanchard and Glasgow’s* ‘*Theoretical model for exercise progression as part of a complex rehabilitation programme design’.(25)* Our progression model (figure 2) uses the unique components of the REACT functional continuum (figure 1). Exercise leaders will use this progression model as a guide to facilitate exercise selection (progression) using examples from the exercise intervention guide (see figure 3A, 3B and 3C).


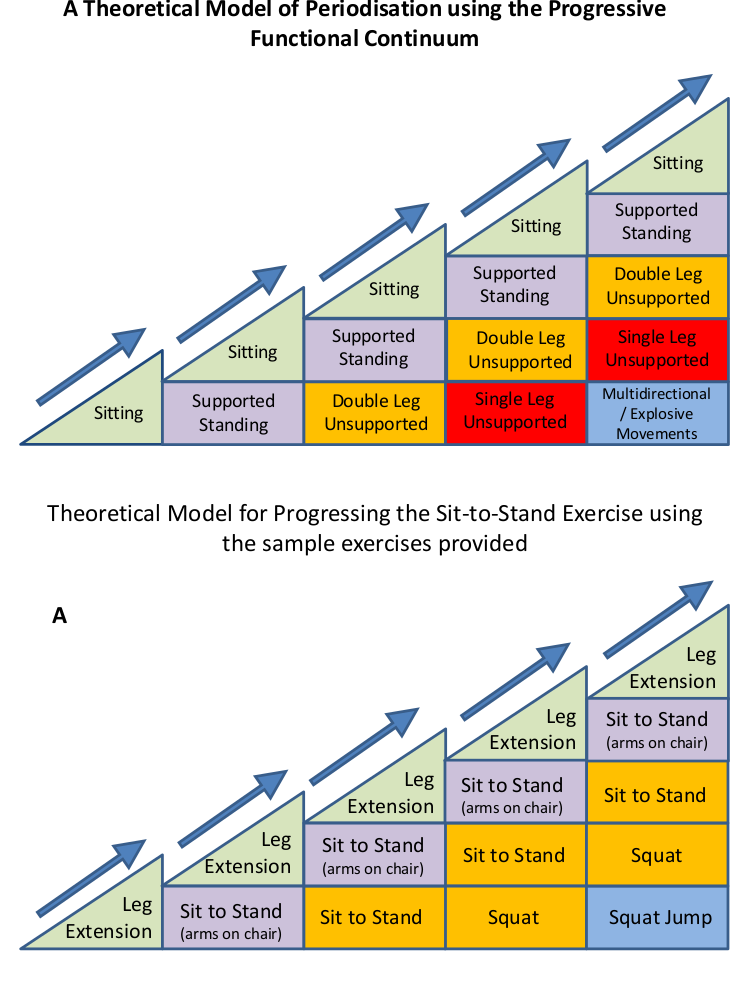


**Figure 2:** Theoretical model of exercise progression using component of the REACT Functional Continuum


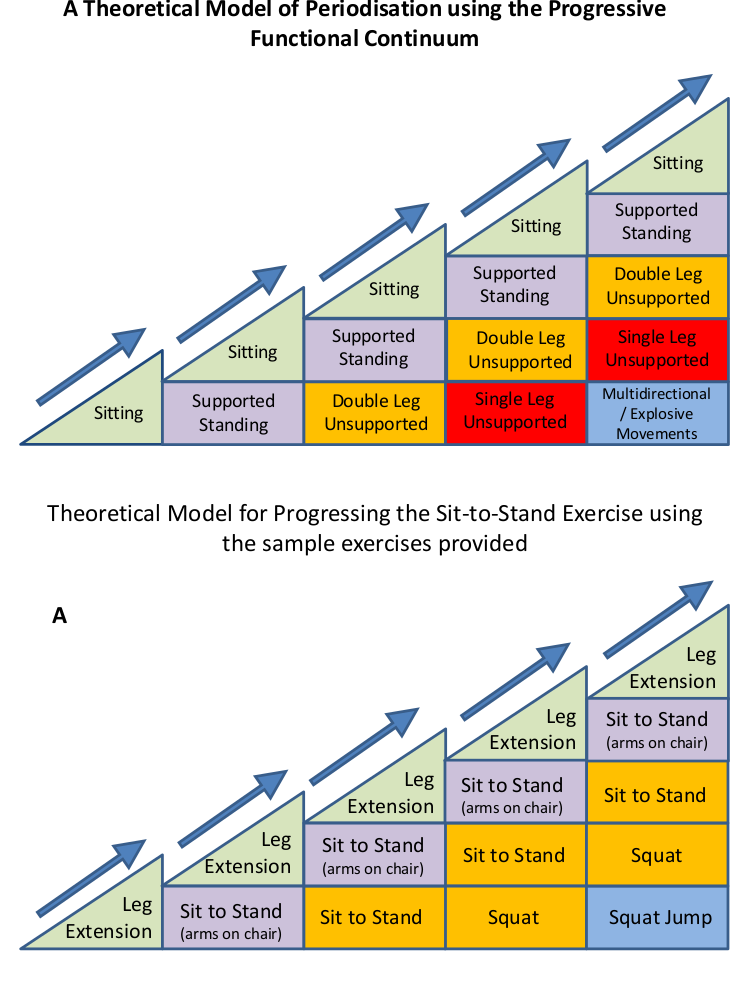

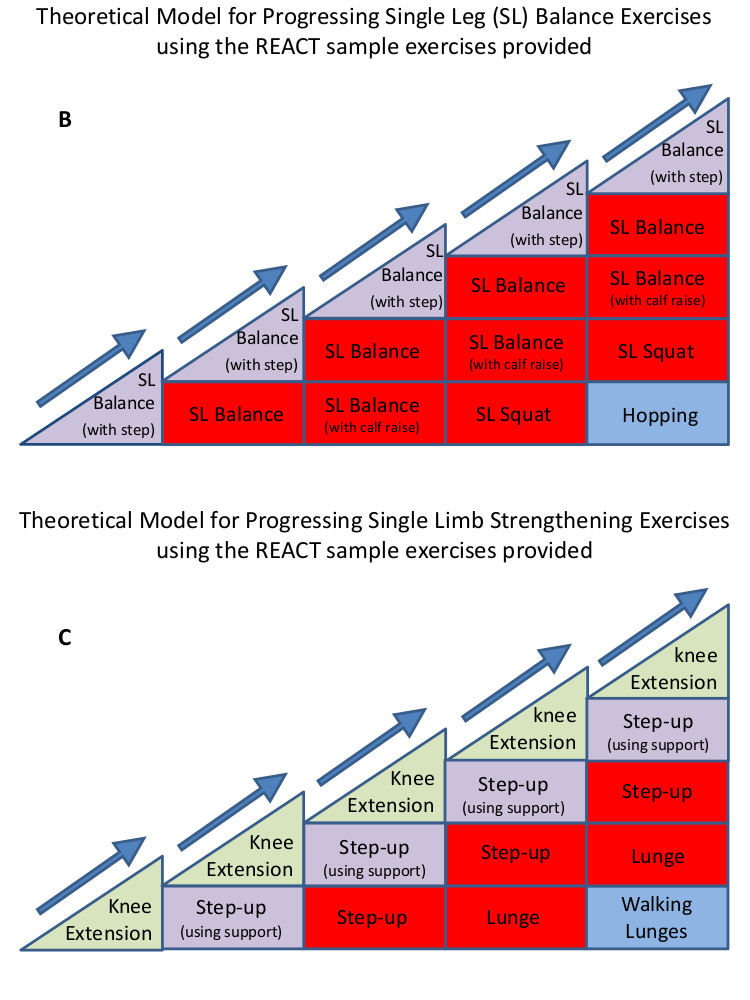


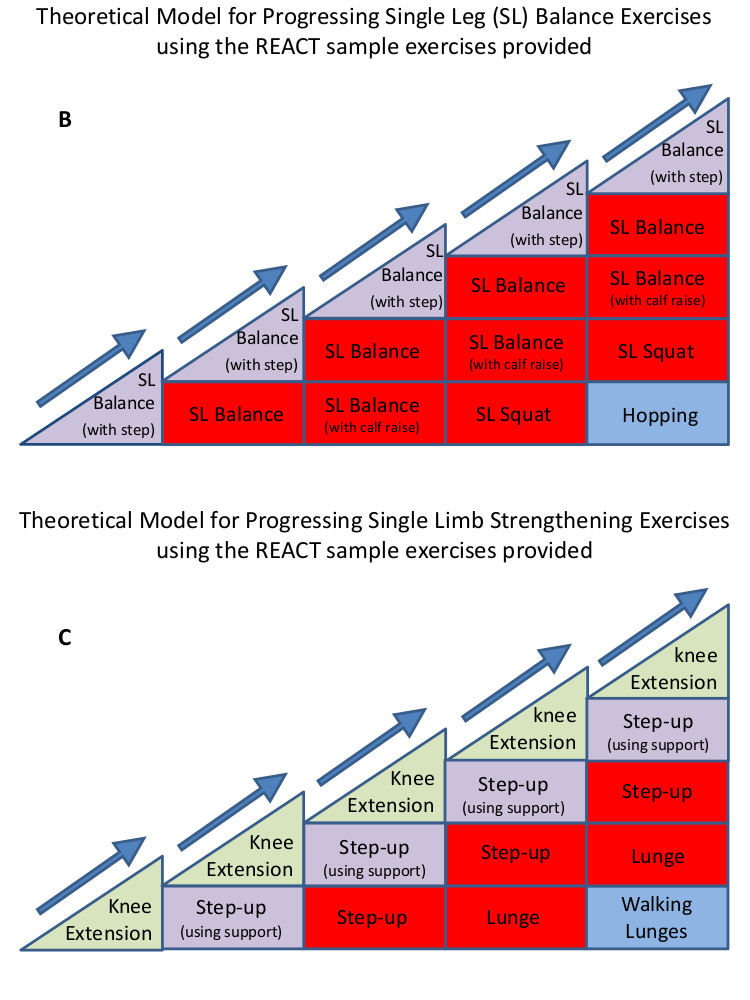


**Figure 3:** Example of how the REACT progression model can be used to progress the development of; (**A**), bilateral lower-limb strength; (**B**), lower limb sensorimotor control (balance); and (**C**), single-limb strength.

The progression models presented here (figure 2 and figure 3) aim to simplify the reasoning process behind the prescription of exercises and may be applied to any given exercise prescribed within the local community settings. The model aims to help exercise leaders visualise the gradual progression of function (figure 2), or of any single exercise (figure 3), and to clinically reason the progressions between exercise sessions. The horizontal axis represents time and the vertical axis the level of functional difficulty. Regarding figure 2, in theory, as the participant adapts to training and becomes more functionally proficient, this increases the number of exercises available to support continual progress and facilitate the individual achieving their functional goals. Each exercise (see figure 3) may be progressed by manipulating a number of variables, including sets, repetitions, speed of contraction, time under tension, base of support, centre of mass, visual aids (eyes open or shut), and the use of external factors (catching an object whilst maintaining balance). The introduction of a new exercise stimulus (new progression), can be interchanged with one another without causing a dramatic progression in the difficulty of the overall exercise. The number and order of exercise progressions is dependent on the individual’s response to exercise and any limiting factors (for example, osteoarthritis causing pain within the joint during specific movements). Therefore, in theory, the number of stages along the horizontal axis and number of progressions along the vertical axis are virtually unlimited and are at the discretion of the REACT exercise leader.

## 3.6.4 Choices and Constraints: Transitioning through the functional continuum as time and physical function progress

When improving ambulatory function is the primary aim of a PA or exercise intervention, limiting group exercise classes to chair-based activities can be considered sub-optimal. In order to observe continual neuromuscular adaptations over a 12 month intervention, a variety of exercise, and therefore opportunities, to add progression are warranted. The one hour of supervised exercise sessions is an ideal opportunity to improve an older adult’s physical competency (mobility, balance, strength and aerobic endurance), confidence and facilitates longer term functional independence. It therefore makes little sense to welcome a group of older adults, who are capable of walking into the exercise session, and ask them to spend the majority of their one hour visit performing activities whilst sat in a chair (unless medically advised). Therefore, a primary objective of the progressive functional continuum is to offer strengthening-based exercises that minimise any reliance on a chair. The REACT exercise protocol facilitates this by using the ‘choices and constraints’ model provided below (figure 4).

*
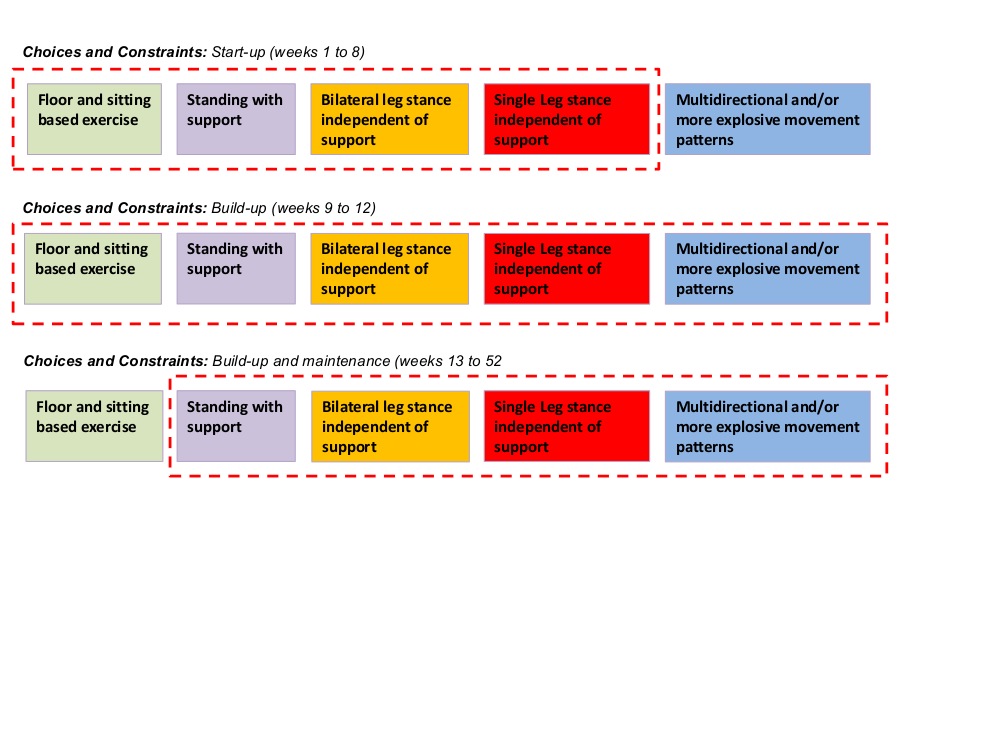
*

**Figure 4:** Diagrammatic description of the choices and constraints to exercise prescription during the 12month REACT study exercise intervention

The choices and constraints model provides exercise leaders with a clear understanding of what type of exercises to prescribe during the supervised sessions during different phases of the overall REACT intervention. Based on the eligibility criteria (section 3.4.1) all participants will be able to walk. Standing based exercises (with and without support) will therefore be encourage from the start of the intervention due to greater transfer to activities of daily living (for example, stair climbing, supermarket shopping, carrying and walking with heavy objects). However, we recognised that not all participants will feel comfortable or confident enough to complete all exercises in standing. Therefore, to optimise enjoyment and adherence during weeks 1 to 8 (start-up), the prescription of chair-based exercises will be permitted alongside standing with and without support (purple, orange and red). During the first 8 week period, multidirectional and or explosive movements will not be permitted. The primary reason for this is that this population are at increased risk of mobility disability with limited history of exercise training experience. Their response to higher impact or functionally challenging movement patterns could be unpredictable and therefore difficult to manage in a large (n=15) group setting. The model reflects this ‘constraint’ between weeks 1 to 8 by excluding the ‘multidirectional and or more explosive movement patterns’ (blue) from within the red dotted box.

During weeks 9 to 12 (build-up) there are no ‘constraints’ with regards to exercise prescription (depicted in the model by all types of exercise being included within the red dotted box). This does not mean that all participants should be doing explosive movements. Only those individuals who have proven during their supervised sessions to be functionally capable of progressing to these higher function tasks (able to perform many of the unsupported standing exercises independently) may start to perform these activities under the supervision of the exercise leader. Exercise prescription will remain at the discretion of the leader, following mutual consent with the participant.

From week 13 onwards (incorporating build-up and maintenance phases) constraints will be placed on chair based exercises (depicted in the model by its exclusion from within the red dotted box). This is to reinforce to the exercise leader that all eligible participants should be capable of performing exercises in a standing position (with support) following 3 months of supervised exercise provision. However, it is important to recognise that an element of common sense is required at all times when supervising exercise delivery of older adults at risk of mobility disability. If a participant feels fatigued or has joint pain during a specific task that can be alleviated by selecting a chair-based exercise, then using a chair should be encouraged. More information is provided in section 3.7.

## 3.6.5 Strategies to promote PA outside of the structured exercise sessions

As a strategy to promote greater engagement in PA outside of the structured exercise sessions, pedometers will be provided to every individual at week 9 as a way of self-monitoring step count. To facilitate greater step counts, at an individual and/or group level, a list of well-known local and national walks will be shared (with their estimated step-counts) (Table 3). Each walk has its own paper-based handout to monitor progress (see example, figure 5). At the end of each day, individuals were encouraged to record their daily step count. At the end of each week they would calculate their weekly step count. This weekly step count number could then be used as a motivation tool for the participant by; 1) providing an objective measure of weekly PA, and 2) by repeating the same walk a few months later and observing their ability to complete the distance (step count) in a much shorter duration of time.

This approach to promote PA enables the exercise leader to observe an individual’s daily ambulatory PA behaviours and identify days within the week where step count is low and starting a conversation about strategies to overcome existing barriers to PA. The monitoring sheet is also a useful tool for the exercise leader to monitor weekly step count and provide weekly goals for each participant to meet (for example, 10% increase in weekly step count).

**Table 3:** List of local/national walks and their step count for monitoring purposes

| **Walk** | **Distance (miles)** | **~ step count** |
| --- | --- | --- |
| Exeter Green Circle | 12 | 24,000 |
| Great North Run | 13.1 | 26,200 |
| Bristol to Bath Railway Path | 13.5 | 27,000 |
| Yorkshire Three Peaks | 24 | 48,000 |
| Lizard peninsula | 61 | 122,000 |
| Hadrian’s Wall | 84 | 168,000 |
| Cotswold Way | 102 | 204,000 |
| Pembrokeshire Coast | 186 | 360,000 |
| Coast-to-Coast | 192 | 372,000 |
| Pennine Way | 267 | 384,000 |

**Please note:** The REACT Leader will not ask the participant to walk the actual footpath, only to increase their daily step count and plot this number of steps on the respective handout sheet.

**
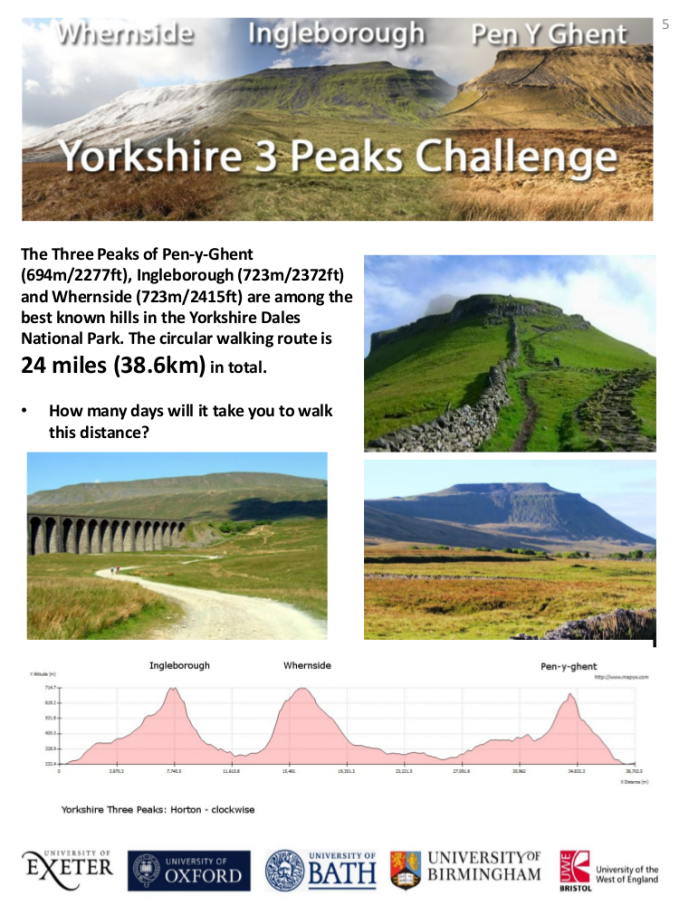

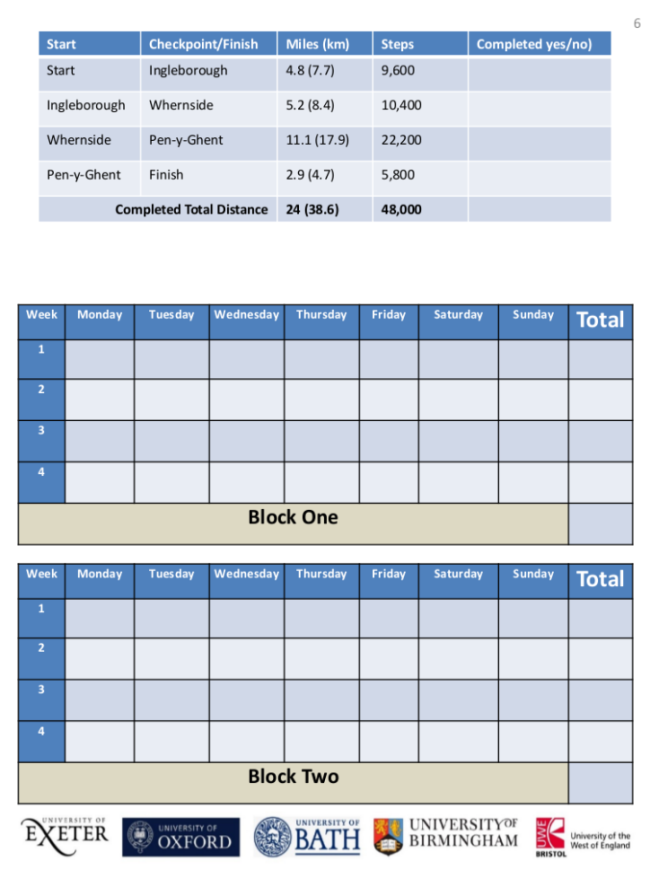
**

**Figure 5:** Yorkshire Dales Three Peak Challenge: step count monitoring sheet

# 3.7 The REACT Study: Personalised Exercise Program

Older adults’ perceptions of their experience in an exercise program are among the major determinants of whether they will continue participation.(26) Previous research has demonstrated that older adults are more likely to continue an exercise program if they find it to be enjoyable.(27, 28)

## 3.7.1 Determining exercise selection for the first exercise session

Due to safety concerns associated with managing a large unknown group for the first time, exercises will be selected primarily from the “standing with support” or ‘chair based’ exercise lists.

During the initial one-to-one introductory session, the exercise leader will observe the individual perform the SPPB. If the individual struggles with their balance and sit to stand tasks (SPPB <6) then chair-based exercises will feature in the first session. However, it is anticipated that most introductory sessions will feature exercises from the ‘standing with support’ based exercise list.

## 3.7.2 Structure of each REACT exercise session

Figure 6 is an example of a single REACT exercise programme, detailing the structure of exercise delivery, exercises selected, number of sets, minimum target of repetitions, ‘target’ RPE (exercise intensity), rest period and ‘actual’ RPE. This example details one of the programmes for that week. An alternative exercise program will be delivered on the second training session, but follow the same structure as outlined below.

Following a progressive 8 to 10-minute warm-up, six strengthening / balance-based exercises will be prescribed to reflect intensities rated ‘moderate to vigorous’ (11 to 16) on a RPE scale. The six exercises will be delivered using a superset method of 3x2 exercise blocks, with 30 to 60 seconds of rest between exercises and a 3-minute rest between each superset block:

Block 1: The first of these exercises will always feature a double limb squat variation (for example, a sit to stand) and an additional lower-limb supplementary exercise (for example, a hip extension)

Block 2: The first of these exercises will always feature a single limb loaded exercise (like a step-up), the second will feature an upper limb exercise alternating between push, pull and press variations (for example a shoulder press).

Block 3: This block of exercise will feature exercises that challenge balancing and rotational control.

Towards the end of each session, games-based activities of 15 to 20 minutes duration will be delivered at ‘light to moderate’ intensities (8 to 13 RPE) and typically incorporated an aerobic conditioning component. Game-based activities will be facilitated via the use of cheap, easily accessible equipment such as bean bags, balls and balloons.

**Figure 6:** Structure of a REACT exercise session

## 3.7.3 Selecting and monitoring exercise intensity

Exercise prescription and progression methods are based on the functional requirements of each individual. Despite being delivered in a group setting, exercise programmes will be personalised to each participant based on their functional status, and using rate of perceived exertion (RPE) methods (a 15-point numerical scale ranging from 6 to 20). (29) By accommodating for daily fluctuations in fatigue and residual muscle soreness, RPE methods encourage more tolerable adjustments to individual training loads on a session-by-session basis,(24) an important consideration for the long-term adherence to any exercise intervention for older adults.(30) Therefore, this individualised approach to exercise prescription enabled each participant to progress at their own pace.

To assist in the decision making process of when an exercise may be considered suitable to progress. The exercise leader will ask the participant how many repetitions in reserve they feel they could produce safely without too much discomfort within that particular exercise. If the participants states more than 3 than modifications to the existing exercise will be introduced (for example, sets, repetitions, speed of contraction, time under tension, base of support, centre of mass, visual aids (eyes open or shut), or the use of external factors (catching an object whilst maintaining balance) or an alternative exercise with a greater functional challenge is introduced

## 3.7.4 Home-based exercise sessions

Three times a week, participants will be encouraged to perform at least 3 exercises at home. This will consist of 2 strengthening based exercises (1 double limb and 1 single limb focused) and one balancing task. The strengthening activities will consist of 3 sets of 12 to 15 repetitions and the balancing task must be attempted at least 3 to 5 times or held for between 30 to 60 seconds. All participants are welcome to do more than this prescribed dose, but exercise selection and dosage will be agreed with the exercise leader beforehand.

A table / chair / kitchen counter can be used to provide a stable surface to assist with balance. These exercises selected are based on mutual decision making between the exercise leader and participant.

# 3.8 The REACT Study: A summary of how the exercise protocol meets national / international PA and exercise guidelines for older adults

The REACT exercise protocol meets national/international guidelines by providing a multimodal exercise intervention designed to improve muscular strength, balance, flexibility and cardiorespiratory fitness. This multimodal exercise program is delivered twice a week for the first 12 weeks in accordance with these guidelines and then reduced to once a week, with the intension of the participant replacing this session with an alternative hour long activity.

Reductions of sedentary time are encouraged by increasing daily step count. Monitoring of daily and weekly step count against well-known UK-based footpaths (figure 5) provide an opportunity to objectively assess increases in ambulatory PA. The exercise protocol also incorporate home-based exercises to be performed a minimum of 3 sessions per week. Subsequently this should encourage individuals to closer meet national PA guidelines of 150 minutes of moderate PA, or 75 minutes of vigorous PA per week

Exercise programmes should be monitored and designed to match the unique physical, psychological, and medical challenges of the individual by following the principles of individualization, periodisation, and progression. To meet this guidelines (in the absence of high cost specialised gym equipment), the ‘functional continuum’ (figure 1), models for progression (figure 2 and 3) and ‘choices and constraints’ models (figure 4) provide clarity on the theoretical framework for multimodal exercise delivery and progression during the 12 month REACT intervention.

# 4.1 Potential implications of findings

If effective and cost-effective, the REACT intervention has strong potential to be implemented widely in the UK and elsewhere. The REACT study is likely to inform UK and international healthy ageing guidance and health promotion policies for the prevention of disability and the maintenance of independent living in older adults.

# 5.1 References

1. Stathi A, Withall J, Greaves CJ, Thompson JL, Taylor G, Medina-Lara A, et al. A community-based physical activity intervention to prevent mobility-related disability for retired older people (REtirement in ACTion (REACT)): study protocol for a randomised controlled trial. Trials. 2018;19(1):1-12.

2. Stathi A, Greaves CJ, Thompson JL, Withall J, Ladlow P, Taylor G, et al. Effect of a physical activity and behaviour maintenance programme on functional mobility decline in older adults: the REACT (Retirement in Action) randomised controlled trial. The Lancet Public Health. 2022;7(4):e316-e26.

3. Snowsill TM, Stathi A, Green C, Withall J, Greaves CJ, Thompson JL, et al. Cost-effectiveness of a physical activity and behaviour maintenance programme on functional mobility decline in older adults: an economic evaluation of the REACT (Retirement in Action) trial. The Lancet Public Health. 2022;7(4):e327-e34.

4. Cross R, Greaves CJ, Withall J, Rejeski WJ, Stathi A. Delivery fidelity of the REACT (REtirement in ACTion) physical activity and behaviour maintenance intervention for community dwelling older people with mobility limitations. BMC public health. 2022;22(1):1-12.

5. Pahor M, Guralnik JM, Ambrosius WT, Blair S, Bonds DE, Church TS, et al. Effect of structured physical activity on prevention of major mobility disability in older adults: the LIFE study randomized clinical trial. Jama. 2014;311(23):2387-96.

6. UK Chief Medical Officers’ Physical Activity Guidelines 2019.

7. Piercy KL, Troiano RP, Ballard RM, Carlson SA, Fulton JE, Galuska DA, et al. The physical activity guidelines for Americans. Jama. 2018;320(19):2020-8.

8. Fragala MS, Cadore EL, Dorgo S, Izquierdo M, Kraemer WJ, Peterson MD, et al. Resistance training for older adults: position statement from the national strength and conditioning association. The Journal of Strength & Conditioning Research. 2019;33(8).

9. Lustosa LP, Silva JP, Coelho FM, Pereira DS, Parentoni AN, Pereira LS. Impact of resistance exercise program on functional capacity and muscular strength of knee extensor in pre-frail community-dwelling older women: a randomized crossover trial. Brazilian journal of physical therapy. 2011;15:318-24.

10. Ryan RM, Deci EL. A self-determination theory approach to psychotherapy: The motivational basis for effective change. Canadian Psychology/Psychologie canadienne. 2008;49(3):186.

11. Marcus BH, Forsyth LH. Motivating people to be physically active: Human Kinetics; 2008.

12. Greaves CJ, Sheppard KE, Abraham C, Hardeman W, Roden M, Evans PH, et al. Systematic review of reviews of intervention components associated with increased effectiveness in dietary and physical activity interventions. BMC public health. 2011;11(1):1-12.

13. Vasunilashorn S, Coppin AK, Patel KV, Lauretani F, Ferrucci L, Bandinelli S, et al. Use of the Short Physical Performance Battery Score to predict loss of ability to walk 400 meters: analysis from the InCHIANTI study. Journals of Gerontology Series A: Biomedical Sciences and Medical Sciences. 2009;64(2):223-9.

14. Lorenz D, Morrison S. Current concepts in periodization of strength and conditioning for the sports physical therapist. International journal of sports physical therapy. 2015;10(6):734.

15. Cadore EL, Izquierdo M. New strategies for the concurrent strength-, power-, and endurance-training prescription in elderly individuals. Journal of the American Medical Directors Association. 2013;14(8):623-4.

16. Janssen I, Heymsfield SB, Wang Z, Ross R. Skeletal muscle mass and distribution in 468 men and women aged 18–88 yr. Journal of applied physiology. 2000.

17. Janssen I, Baumgartner RN, Ross R, Rosenberg IH, Roubenoff R. Skeletal muscle cutpoints associated with elevated physical disability risk in older men and women. American journal of epidemiology. 2004;159(4):413-21.

18. Hardy SE, Kang Y, Studenski SA, Degenholtz HB. Ability to walk 1/4 mile predicts subsequent disability, mortality, and health care costs. Journal of general internal medicine. 2011;26(2):130-5.

19. Reid KF, Fielding RA. Skeletal muscle power: a critical determinant of physical functioning in older adults. Exercise and sport sciences reviews. 2012;40(1):4.

20. Ramírez-Campillo R, Castillo A, Carlos I, Campos-Jara C, Andrade DC, Álvarez C, et al. High-speed resistance training is more effective than low-speed resistance training to increase functional capacity and muscle performance in older women. Experimental gerontology. 2014;58:51-7.

21. Straight CR, Lindheimer JB, Brady AO, Dishman RK, Evans EM. Effects of resistance training on lower-extremity muscle power in middle-aged and older adults: a systematic review and meta-analysis of randomized controlled trials. Sports medicine. 2016;46(3):353-64.

22. Tschopp M, Sattelmayer MK, Hilfiker R. Is power training or conventional resistance training better for function in elderly persons? A meta-analysis. Age and ageing. 2011;40(5):549-56.

23. Blanchard S, Glasgow P. A theoretical model for exercise progressions as part of a complex rehabilitation programme design. BMJ Publishing Group Ltd and British Association of Sport and Exercise Medicine; 2019. p. 139-40.

24. Buskard AN, Jacobs KA, Eltoukhy MM, Strand KL, Villanueva L, Desai PP, et al. Optimal Approach to Load Progressions during Strength Training in Older Adults. Medicine and science in sports and exercise. 2019;51(11):2224-33.

25. Blanchard S, Glasgow P. A theoretical model to describe progressions and regressions for exercise rehabilitation. Physical therapy in sport : official journal of the Association of Chartered Physiotherapists in Sports Medicine. 2014;15(3):131-5.

26. Brassington GS, Atienza AA, Perczek RE, DiLorenzo TM, King AC. Intervention-related cognitive versus social mediators of exercise adherence in the elderly. American journal of preventive medicine. 2002;23(2):80-6.

27. Rhodes RE, Martin AD, Taunton JE, Rhodes EC, Donnelly M, Elliot J. Factors associated with exercise adherence among older adults. Sports medicine. 1999;28(6):397-411.

28. Thompson CE, Wankel LM. The effects of perceived activity choice upon frequency of exercise behavior. Journal of Applied Social Psychology. 1980;10(5):436-43.

29. Borg GA. Psychophysical bases of perceived exertion. Medicine and science in sports and exercise. 1982;14(5):377-81.

30. Chodzko-Zajko WJ, Proctor DN, Singh MAF, Minson CT, Nigg CR, Salem GJ, et al. Exercise and physical activity for older adults. Medicine & science in sports & exercise. 2009;41(7):1510-30.
